# Systematic Computation of Nonlinear Cellular and Molecular Dynamics with Low-Power CytoMimetic Circuits: A Simulation Study

**DOI:** 10.1371/journal.pone.0053591

**Published:** 2013-02-05

**Authors:** Konstantinos I. Papadimitriou, Guy-Bart V. Stan, Emmanuel M. Drakakis

**Affiliations:** 1 Department of Bioengineering of Imperial College, South Kensington Campus, London, United Kingdom; 2 Centre for Synthetic Biology and Innovation, Imperial College, South Kensington Campus, London, United Kingdom; University of Minnesota, United States of America

## Abstract

This paper presents a novel method for the systematic implementation of low-power microelectronic circuits aimed at computing nonlinear cellular and molecular dynamics. The method proposed is based on the Nonlinear Bernoulli Cell Formalism (NBCF), an advanced mathematical framework stemming from the Bernoulli Cell Formalism (BCF) originally exploited for the modular synthesis and analysis of linear, time-invariant, high dynamic range, logarithmic filters. Our approach identifies and exploits the striking similarities existing between the NBCF and coupled nonlinear ordinary differential equations (ODEs) typically appearing in models of naturally encountered biochemical systems. The resulting continuous-time, continuous-value, low-power CytoMimetic electronic circuits succeed in simulating fast and with good accuracy cellular and molecular dynamics. The application of the method is illustrated by synthesising for the first time microelectronic CytoMimetic topologies which simulate successfully: 1) a nonlinear intracellular calcium oscillations model for several Hill coefficient values and 2) a gene-protein regulatory system model. The dynamic behaviours generated by the proposed CytoMimetic circuits are compared and found to be in very good agreement with their biological counterparts. The circuits exploit the exponential law codifying the low-power subthreshold operation regime and have been simulated with realistic parameters from a commercially available CMOS process. They occupy an area of a fraction of a square-millimetre, while consuming between 1 and 12 microwatts of power. Simulations of fabrication-related variability results are also presented.

## Introduction

The human body can be viewed as an incredibly complex biological oscillator that exhibits prominent harmony between all cellular rhythms in it, thanks to the enviably efficient energy and performance properties of the cells. With an average net power consumption of only 1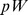, performance of approximately 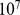 ATP-dependent biochemical reactions per second and typical dimensions that do not exceed 10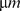, the average human cell is undoubtedly an unmatched “*biological microprocessor*” of various types of signals [1], [2].

Although cells are accurate and power-efficient “*biological processors*”, in most cases they require specific conditions and a certain amount of time from start to completion of an operation. For example, one of the most important cellular oscillations in the human body, mitosis, is a highly demanding procedure, which undergoes several stages and requires a large period of time, usually several hours, until it is completed [1], [3]. In addition, even small changes in experimental parameters of a biological process implemented *in vitro* might lead to significant phenotypic variations and require repetition of the whole process, leading to loss of valuable test time and ultimately to high cost.

For these reasons, it can be argued that it is very advantageous to simulate biological and biochemical dynamics by means of powerful computers, which use precise and accurate numerical simulation methods and are able to process huge amounts of data, based on the mathematical equations that describe each cellular or molecular function. Various reduced or extended mathematical models have been proposed, particularly during the last few decades, defining in a more or in a less accurate mathematical way most of the biological rhythms, which take place in the human cell. More specifically, the mathematical description of cellular behaviour has progressed to such a level that a gene-protein regulation network or a cellular/neural network can now be efficiently described by a system of coupled nonlinear differential equations, which incorporate properties, such as stochasticity and cell variability [4]–[6].

Albeit the mathematical models describing cellular functions have reached an adequate level of accuracy and can be simulated with the use of powerful software, when it comes to the simulation of very large networks of cells, whose dynamics include nonlinearity, stochasticity, cell variability, dynamic uncertainties and perturbation, software simulations start to become extremely demanding in computational power [2]. Moreover, computer simulations are not always suitable for human-machine interaction, since continuous monitoring might be required in conjunction with small device area and low power consumption.

This appearing gap that exists between computer simulations and biology can be filled with the use of certain biomimetic engineering devices, which are capable of generating dynamical behaviours similar to the biological ones observed experimentally. With the use of ultra-fast, ultra-low-power analog chips that are able to simulate single or multiple cell operations and are organised in highly parallel formation, it is possible to implement large VLSI cell networks, which - in principle - could include the time-varying stochastic parameters that define a biochemical system [7].

The striking similarities between the equations describing biochemical systems and the equations defining the current-voltage relations between properly interconnected subthreshold MOS devices and capacitors, provide the motivation to emulate a real life cellular behaviour by means of an ultra-low power electrical circuit. The potentials of such an endeavour are tremendous: with the use of the aforementioned circuits, researchers would be able not only to simulate biological responses fast and accurately by simply altering different biological parameters that can be translated into certain electrical parameters, but would also be able to predict a future cell behaviour following a deterministic or a stochastic dynamical description.

Inspired by the above, the aim of this paper is to introduce a systematic way of designing such electrical circuits by exploiting the similarities between the Nonlinear Bernoulli Cell Formalism (NBCF) and systems of ordinary differential equations (ODEs) that characterise biochemical processes. The flexibility provided by the NBCF allows us to use simple static translinear blocks for the implementation of mathematical operations, in combination with dynamic translinear blocks whose current-voltage logarithmic behaviour is characterised by the Bernoulli differential equation, to realise in full the differential equations, which specify the considered biological systems.

The paper is structured as follows: Firstly, we introduce the biological models that characterise the cellular and molecular behaviours. Then present the log-domain mathematical framework used for the transformation of the biological equations into the electrical ones. To illustrate the striking similarities between the original equations and the electrical ones, an in depth mathematical analysis is provided exhibiting the nonlinear properties of both models and examining how close these models are to each other. After the mathematical treatment of both models, a section comparing simulations of these dynamical models produced by MATLAB^©^ and Cadence software platforms is presented. Moreover, a section investigating the robustness of the proposed circuits based on Monte Carlo Analysis and Transient Noise Analysis simulations follows. Finally, a discussion section is presented commenting on the similarities of both biological and electrical models and providing an insight into the envisaged applications of such bioinspired devices.

## Modelling Intracellular Signals

Cells in multicellular organisms need to communicate with each other during their daily functions, in order to accomplish a large number of operations, such as cell division, apoptosis or differentiation. The remarkable ways through which this communication is achieved is a result of complicated combinations of electrical or chemical signalling mechanisms. This paper focuses on one of the key intracellular signalling processes, the intracellular calcium (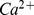) oscillations [1]. Analysing the background mechanisms leading to the oscillatory behaviour of intracellular 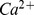 and presenting the mathematical models proposed for the description of these oscillations, we aim at demonstrating a systematic approach for the design of VLSI circuits that are able to generate similar dynamics to the ones produced through the aforementioned intracellular signalling processes.

### Models of intracellular calcium oscillations

Being amongst the most important cellular rhythms in the field of biological oscillations and body rhythms in general, 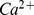 oscillations exhibit great interest for a plethora of reasons. Apart from the fact that 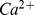 oscillations occur in a large number of cells either spontaneously or after hormone or neurotransmitter stimulation, these rhythms are often associated with the propagation of 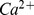 waves within the cytosol and neighboring cells [1]. Moreover, the undisputable regulatory properties of 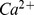 in a wide range of cell operations, such as metabolic/secretory processes, cell-cycle progression, replication or gene expressions combined with the vast number of cell types, where 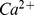 oscillations take place in, (e.g. cardiac cells [8], oocytes, hepatocytes [9], endothelial cells [10], fibroblasts or pancreatic acinar cells) underline the importance of this intracellular signal and stress the need for the development of accurate mathematical models that can efficiently describe this type of intracellular oscillation [1].

Due to the Poincaré
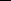
Bendixson theorem [11] at least a two-variable system of kinetic equations is required for the realisation of self-sustained oscillations. As illustrated in [12], at least five minimal models can be conceived for this biochemical type of oscillation. Apart from the two-dimensional model proposed by Goldbeter and his collaborators [13], a focal point of this paper, other minimal models such as the ones presented by Li and Rinzel [14] and Marhl *et al.* [15] can be used to describe this intracellular rhythm, each one exploiting a different system process, such as 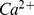 exchange with extracellular medium, inositol triphosphate receptor (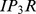) desensitisation or even 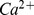 binding to proteins [12]. In the following paragraphs, a brief analysis will be presented regarding the prevalent, experimentally verified mechanism for 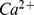 oscillations in cells.

#### Models For 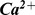 Oscillations Based On 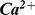-Induced 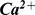-Release Mechanism

According to a feedback mechanism proposed by Berridge [16], [17], 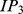 triggers 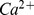 mobilisation from an intracellular store causing cytosolic 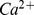 to be transported into an 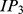-insensitive store from which it is released in by a 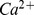 activated process [1]. This mechanism, which has been experimentally demonstrated in the past, is also known as “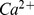 *-Induced* 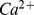 *-Release*” mechanism or *CICR*. The existence of this specific intracellular mechanism has been verified in a wide variety of cells [1].

By taking the principles of the aforementioned “*structure*” into consideration, Goldbeter and his collaborators [1], [13], [18]–[22] developed a reduced and an extended model, which accurately and efficiently describe 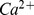 oscillations. Relying on the hypothesis that the amount of 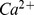 released is controlled by the level of stimulus through modulation of the 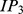 level and by making the simplification that the level of stimulus-induced, 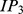-mediated 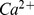 is a model parameter, the following two-dimensional minimal model for the description of intracellular 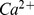 oscillations is generated:
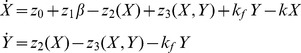
(1)with
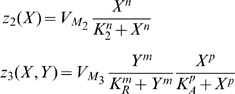
The quantities X and Y denote the concentration of free 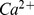 in the cytosol and in the 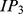-insensitive pool, respectively. Moreover, 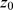 denotes the constant 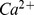 input from the extracellular medium and 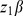 refers to the 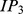-modulated release of 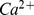 from the 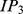-sensitive store. The parameter 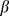 defines the amount of 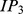 and therefore measures the saturation of the 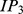 receptor [1]. The values of 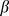 typically range from 0 to 1. The biochemical rates 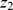 and 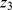 refer, respectively, to the pumping of 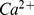 into the 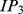-insensitive store and to the release of 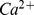 from that store into the cytosol. The parameters 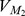, 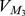, 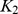, 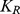, 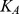, 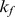 and 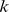 are the maximum values of 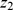 and 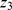, threshold constants for pumping, release and activation and rate constants, respectively [1], . It is worth mentioning that the dimensions of the quantities in (1) are 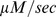.

A major advantage of the above two-dimensional model is the flexibility that it provides regarding the selection of the cooperativity factors. Parameters 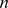, 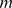, and 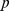 define the Hill coefficients characterising the pumping, release and activation processes, respectively. Depending on the values of the Hill coefficients, different degrees of cooperativity can be achieved and this consequently allows us to study different cellular functions. For example, in this type of intracellular signaling, pumping is known to be characterised by a cooperativity index 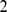 [23]. However, higher degrees of cooperativity have also been observed experimentally [1] [19].

Three different cases of Hill coefficients have been investigated for the purposes of this paper. Based on [1], [13], [18]–[22] the case of 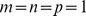, which corresponds to non-cooperative behaviour is treated first. Subsequently, we consider the case where 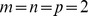 and conclude with the 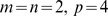 case, which implies high activation cooperativity. All three cases have been simulated by means of MATLAB^©^ simulations and realised by means of new, ultra-low-power analog circuits. The fact that the model is two dimensional makes it suitable for extended phase plane analysis, based on the Poincaré
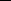
Bendixson theorem.

## Modelling Genetic Regulatory Systems

In the 2002 paper of Chen and Aihara [24], a gene-protein regulatory system was proposed and modelled by a nonlinear system of coupled differential equations. It is a gene system with an autoregulatory feedback loop, which can generate periodic oscillations for a specific number of parametric values. The biomedical application of the proposed multiple time scale model is that it can act as a genetic oscillator or even as a switch in gene-protein networks, due to the robustness of the dynamics produced for different parameter perturbations [24]. This elegant nonlinear system can be also used for the qualitative analysis of periodic oscillations, such as circadian rhythms, which appear in most living organisms with day-night cycles. Similar network models have been proposed in [25] and [26], all of them aiming to contribute to the establishment of new biotechnological design methods [24]. Chen and Aihara's model is described by the following two-dimensional set of coupled nonlinear differential equations:
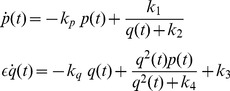
(2)where 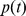 and 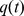 express time-dependent protein concentrations, 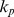 and 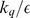 are degradation rates, 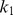 is the transcription and translation rate for gene P, 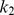 is the Michaelis-Menten constant and 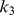 and 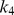 are lumped parameters, describing the binding, multimerisation of protein and phosphorylation effects [24]. The quantity 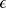 is a real, positive number controlling time scaling.

In addition, in the same paper, a three dimensional biologically plausible model has been presented, in order to verify their initial assumptions. In this model, proteins 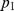 and 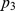 form a heterodimer, which inhibits expression of 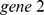, while protein 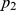 forms another heterodimer for the activation of 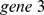 and simultaneous inhibition of 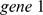. The aforementioned process is described by the following set of three nonlinear coupled differential equations:
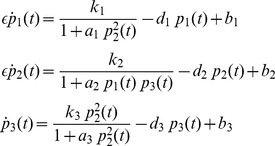
(3)This model is based on the assumption that the production of proteins 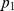 and 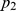 takes place much faster than the production of 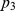. The remaining quantities of the three dimensional model are appropriate biological kinetic parameters. The quantities in (2) and (3) have no units, due to lack of experimental data [24].

## Mathematical Framework

### The Bernoulli Cell formalism: A MOSFET type-invariant analysis

**Figure 1:**
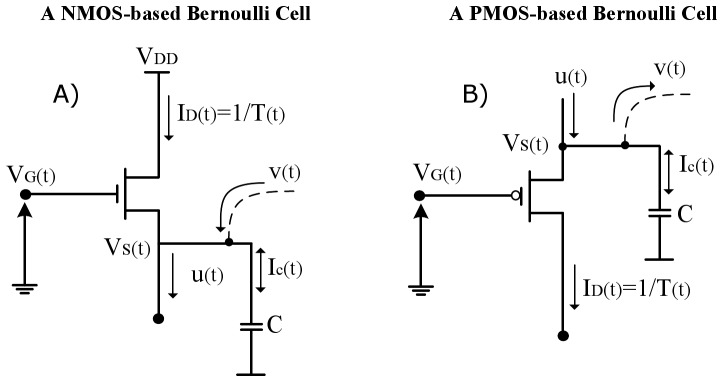
A NMOS and PMOS based Bernoulli Cell. The arrows defining the direction of the capacitor current are bidirectional, since the BC analysis holds, whether the capacitor is connected to ground or 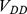.

The term Bernoulli Cell (BC) was coined in the international literature by Drakakis in 1997 [27] in an attempt to describe the relation governing an exponential transconductor and a source-connected linear capacitor, whose other plate is held at a constant voltage level (e.g. ground). It has been shown that the current relation between these two basic monolithic elements is the well known Bernoulli differential equation. As Figure 1 illustrates, by setting the drain current as the state variable of our system and by means of a nonlinear substitution (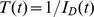), we can express the nonlinear dynamics of the BC in a linearised form.

The current relation of an NMOS device operating in weak-inversion [28] is described by the following relation:
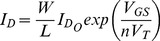
(4)where 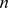 is the subthreshold slope factor, 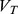 is the thermal voltage (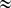
26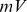 at 300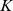), 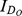 is the leakage current of the transistor and W, L are the width and length of the device, respectively. Assuming 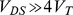, the factor of the complete weak-inversion drain current relation shown in [28], 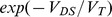, can be omitted.

Based on (4), the drain currents of the NMOS and PMOS transistors can be re-expressed as follows, taking into consideration their nonlinear substitution and setting 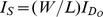:
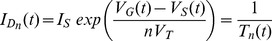
(5)

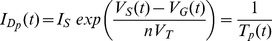
(6)By differentiating (5) and (6) with respect to time:
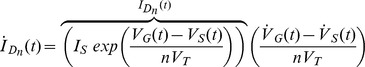


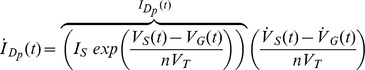

Figure 1 shows that in the case where the bottom plate of the capacitor is held at ground, application of Kirchhoff's Current Law (KCL) provides the following relations for both cases:
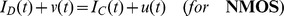


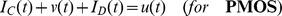
where 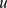 and 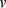 are defined as the input and output currents of the BC. Similar analysis holds if the bottom plate of the capacitor is held at 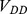.

**Figure 2:**
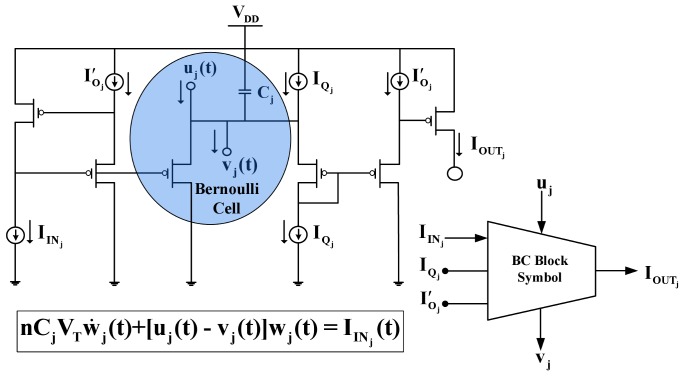
Schematic and symbolic representation of the dynamic TL block, which “*hosts*” the Bernoulli Cell. All devices have the same W/L ratio.

By substituting the current expressions derived from KCL into the aforementioned drain current differential equations, we end up with the following set of differential equations for both transistor types:
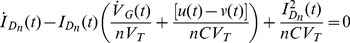
(7)

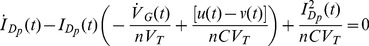
(8)The form of (7) and (8) comply with the Bernoulli differential equation and by substituting 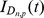 with 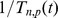 (and consequently 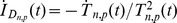) :
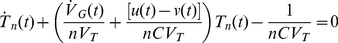
(9)

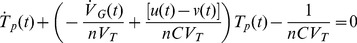
(10)Driving both devices by a logarithmically compressed input current (see Figure 2) so that 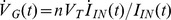 and 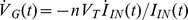 for the NMOS and PMOS case, respectively, yields:
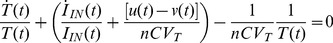
(11)or equivalently to
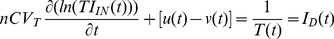
(12)for both types of MOSFETs.

From (12), defining a new dimensionless state-variable 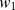, which is defined as 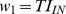, we end up with the following final expression:
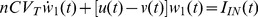
(13)By connecting 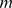 BCs in series (“*cascade*” topology), where the gate voltage of the first one is logarithmically driven by a constant input current 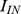 (see Figure 2), while the gate voltage of the rest BCs is controlled by the capacitor variations of the previous BC, a set of generic dynamics termed Log-Domain-State-Space (LDSS) is generated [29]. The LDSS relations are simply the linearised differential equation expressions of the nonlinear differential equations governing the corresponding BC and have the following form:
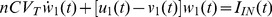
(14a)

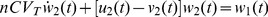
(14b)

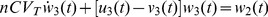
(14c)

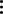


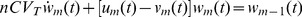
(14d)where the subscript 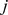 (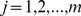) corresponds to the 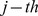 BC of the cascade, while the variables 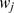 are defined as follows:
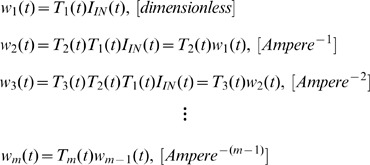
(15)The derivation of (14.b), (14.c) etc. follows a procedure identical to the one explained before.

For Externally-Linear, Internally-Nonlinear (ELIN) applications [30], such as the synthesis and the analysis of log-domain filters [29], [31], the usefulness of this formalism is that it bypasses the nonlinearity of log-domain dynamics by converting them into their linearised equivalent form [27], [29], [32]. However, the BCF, or more specifically a new, modified version of it, termed Nonlinear Bernoulli Cell Formalism (NBCF) can be used for non-cascaded BCs as well. Instead of selecting to connect in tandem 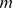 single BC hosting log-domain integrator-like translinear (TL) circuits, where the current output of the previous one becomes the current input to the next one [29], single, independent dynamic translinear blocks can be connected together (say 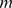 again in number) with their inputs and outputs connected in a coupled way (“*coupled*” BC topology). As will be shown later, it is the coupled interconnection of the dynamic translinear blocks, which “*host*” the BCs that will allow us to implement the coupled nonlinear biological differential equation systems.

Starting from the fact that each differential equation of the LDSS can exist independently, a sub-category of the LDSS can hold for 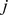 in number dynamic translinear blocks, each described by the following equation:
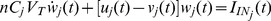
(16)with
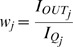
(17)where 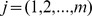, 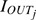 is the output current of the 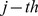 BC, while 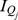 is the shifter current of the 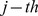 TL circuit (see Figure 2), which “*hosts*” the BC.

The careful selection of the input and output currents 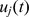, 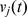 and 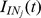 of the BC allows us to construct various types of differential equations (linear or nonlinear) and consequently implement them by means of an analog circuit. The appropriate selection of these BC currents is dictated by the targeted biochemical dynamics. Thus, their systematic realisation is leading to the generation of the new type of circuits, termed CytoMimetic circuits.

## Synthesis Method of Analog CMOS CytoMimetic Circuits

In the previous section of the paper, the term CytoMimetic circuits was introduced. This distinct class of bioinspired circuits aims at simulating cellular and molecular dynamics, based on the mathematical expressions of various, nonlinear, biological models. Our attempts on implementing a wide range of nonlinear models so far, show that the NBCF formalism is a useful tool for transforming biochemical models into their electrical equivalent and as a result design analog circuits, whose outputs will produce dynamics that are very close to the ones of the prototype systems.

More specifically, the scope of CytoMimetic circuits is to mimic the time-dependent behaviour of biochemical substances as they are observed experimentally, relying on a time-scaled approach. Thus, there is a distinct difference between them and the other categories of bioinspired circuits, e.g. Neuromorphic [33]–[35], which mainly focus on circuits that simulate biological dynamics related to electrical activities of the cell. In contrast to the Neuromorphic case, the intrinsic nonlinear cellular and molecular dynamics that CytoMimetic circuits realise relate with the dynamical behaviour of biochemical quantities, whose concentration is strictly positive.

The direct correspondence between electrical and biological variables and parameters stemming from the NBCF provides the flexibility required for the realisation of various nonlinear mathematical models by computing their time-dependent dynamical behaviour. The following paragraphs present the method through which we migrate from the biological to the electrical field of equations and will offer a systematic methodology to approach nonlinear biochemical models.

### Building the general form of the electrical analogous equations

The basic structure of the electrical analogous equations is provided by (16) and (17) and is physically implemented by the BC block presented in Figure 2. This form of equations creates the starting transistor-level scaffold, on which the electrical equivalent system can be built. The counterintuitive, dimensionless parameters 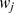 of the linearised BCF serve as the new variables of the electrical model, which map the biological model's variables onto the electrical equations system. For the implementation of a 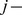
dimensional nonlinear equation system it is clear that 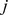 BC blocks need to be used, each one corresponding to a different biological variable of the prototype model. Therefore, (16) can be generalised and in theory one can have a 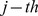 order LDSS described by the following equations:
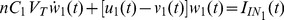
(18a)

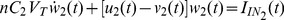
(18b)

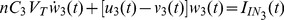
(18c)

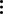


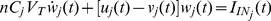
(18d)It should clear that (18) introduces a specific form of LDSS, suitable for the description of coupled linear/nonlinear systems with the coupling realised through the dependence of the 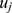, 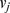 and 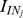 currents on other 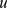, 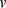 and 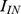 currents. The major difference between (14) and (18) lies in the RHS of the equations. For the LDSS equations (14) the RHS of all equations, except for the first one, is a function of 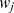, due to the cascaded topology, where the input of the next BC is the output of the previous one (except for the 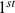 BC) [27], [29]. On the other hand, for the RHS of (18), it is convenient that one can taylor the input as a function of the 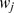 variables in a manner dictated by the targeted dynamics. The coupled BC topology - as opposed to the cascaded one - provides the flexibility to use the NBCF in various types of nonlinear differential equations, including the ones presented in (1), (2) and (3). It should be borne in mind that in this case the variable 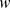 is dimensionless. It is the mapping of the biological parameters onto the dimensionless 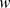 that helps us maintain unit consistency in the electrical equivalent equations.

Now it is time to explain how one can define the input and output currents of the NBCF, which will help us complete the formation of the electrical equations. Being implemented by static TL blocks, the input/output currents 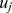 and 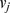 of the BC may become a function of other variables and/or other input currents, e.g.
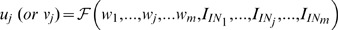
or simply adopt constant values, i.e. 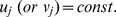


However, the selection of the appropriate 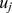 and 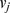 currents in each BC TL block consists the major challenge of the synthesis phase of CytoMimetic circuits. The choice of which factors of the ODE should correspond to the input/output currents of the BC might become easier when re-expressing the target nonlinear ODE in the form of (16) or (18).

By separating the terms of the ODE - which are a function of the equation's variables - from the other terms, presenting them onto the LHS of the equations and then setting the system's variables as a common factor, will eventually generate a form similar to (16) or (18). The exemplary, fictitious, two-dimensional system of nonlinear equations (19) and (20) provide an example of the above methodology. Let it be assumed that the following biochemical dynamics are targeted:
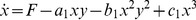
(19)

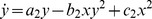
Expressing (19) in a form similar to (18):
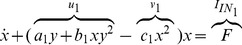
(20)

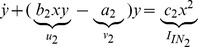
where 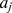, 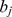, 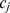, 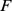 (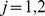) are constants of appropriate dimensions so that dimensional consistency of (19) and (20) is preserved.

Following this treatment, the terms inside the parenthesis on the LHS may be treated as the 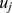 and 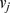 currents of the 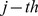 BC, depending on the sign of the terms. However, such an approach though correct mathematically might not always lead to the desirable, practical results. Practical electrical constraints must be also taken into consideration. In particular, effort should be put into ensuring that for the anticipated current value range - which in practice is determined by the form of the targeted biological dynamics - the devices remain in the subthreshold regime, which in turn ensures the validity of the LDSS.

Exploiting the freedom provided by NBCF a mathematical equation can be expressed into various equivalent electrical ones; we opt to select the electrical analogous model, which not only implements the desired biological model dynamics but also facilitates compliance with the subthreshold region constraints of MOS operation.

### Electrical circuit blocks

CytoMimetic circuits comprise medium complexity dynamic and static TL circuits. Although the majority of the mathematical models that describe cellular or molecular behaviour might require a wide range of different TL blocks combinations, most of them could be derived from or would be a combination of three basic blocks, given that various mathematical operations could be also implemented using different TL network realisations. Regardless of the TL combination chosen to generate the required mathematical operations, the NBCF will hold. In order to demonstrate the systematic nature of the proposed framework in this paper, the following TL blocks have been used for the implementation of all five electrical equivalent circuits presented in this work.

#### The BC block

The BC block presented in Figure 2 is responsible for generating the general form of the electrical equivalent equations, described by (16) and (18). By being the TL block, which “*hosts*” the Bernoulli Cell, it provides an output current 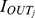, which emulates one of the time-dependent variables of the prototype biochemical model.

#### The squarer block

**Figure 3:**
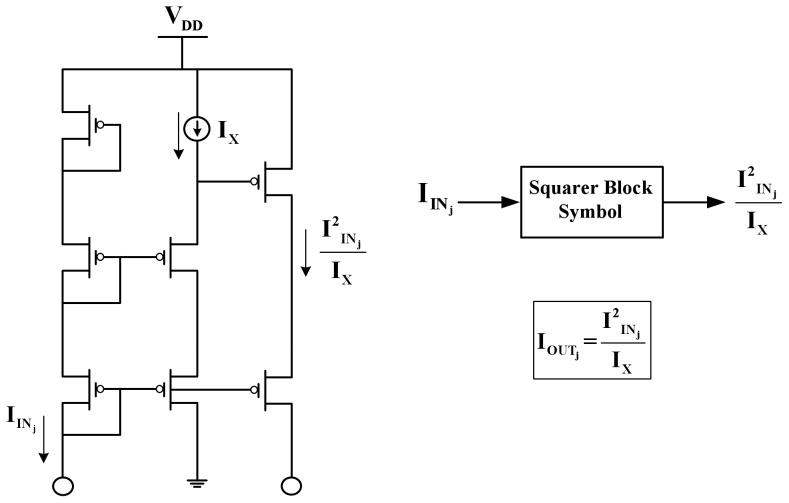
Schematic and symbolic representation of the squarer TL block. All devices have the same W/L ratio.

With all devices having the same W/L ratio, the squarer block of Figure 3 produces the square of an input current over a scaling current, expressed as 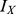 in our circuits. Without loss of generality, the scaling current usually has the value of 1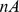, so that the numerical squared value of the input current is received at the circuit's output. A cascoded topology has been selected to minimise output current errors.

#### The multiplier/divider block

**Figure 4:**
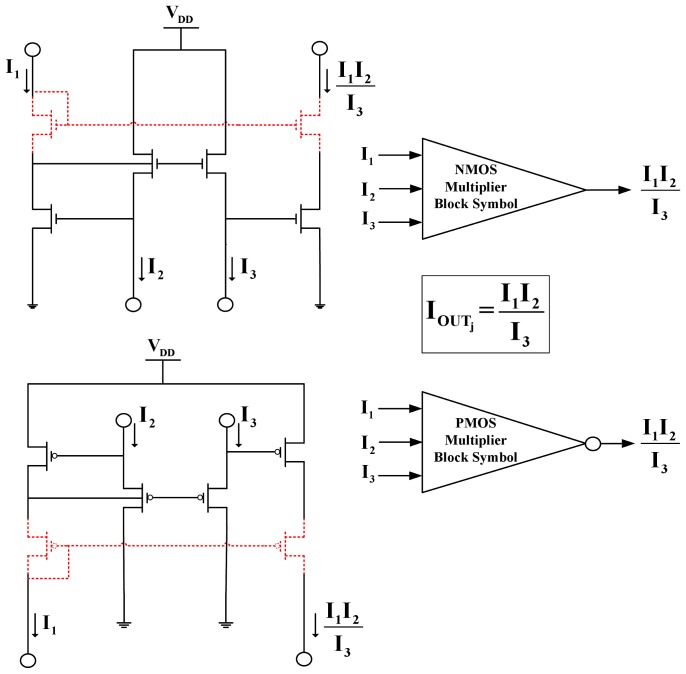
Schematic and symbolic representation of the multiplier/divider TL block. Note that both blocks presented in this Figure are cascoded TL blocks. Depending on the accuracy required for each application, CytoMimetic circuits can operate with non-cascoded multiplier TL blocks. The symbolic representation for the non-cascoded multiplier is similar to the one presented here but with a star placed inside the symbol (see for example Figure 6). In the non-cascoded topology, the devices that are sketched with dashed lines are absent.

Employing devices of the same W/L aspect ratio, the multiplier block allows us to perform multiplication or division operations with currents based on the TL principle: 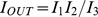 (see Figure 4). Again, cascoded topologies have been selected to minimise output current errors.

## Example Synthesis of Two Biochemical Systems

From (1), (2) and (3), five mathematical models can be derived, each one implementing a biological/biochemical function with different properties. In this paper we opt to present in detail the synthesis procedure leading to the electrical equivalent equations and circuits for two prototype models, one from each category. Thus, for the intracellular 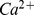 oscillations model, the case where the Hill coefficients 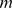, 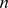, 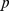 are equal to two has been selected, while for the gene-protein regulatory models the two-dimensional case will be elaborated. It is important to mention that the remaining categories of models have been also analysed in a similar way. However, owing to lack of space, it has been decided not to describe and detail the transformation of all prototype equations into their electrical equivalent circuits though confirming simulation results are presented for all cases.

At this point it must be stressed that regarding the time properties of the implemented electrical analogous circuits, a nonlinear dynamical system approach should be adopted, in order to estimate - roughly - the frequency of oscillation of the considered electrical systems [11], [36]–[39]. Contrary to the case of input-output linear log-domain circuits and although the quantities 
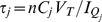
 (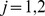) have dimensions of *seconds*, they should not be associated to the nonlinear systems' frequency of oscillations. Such quantities now relate to the time scaling of the CytoMimetic electrical equivalents.

**Figure 5:**
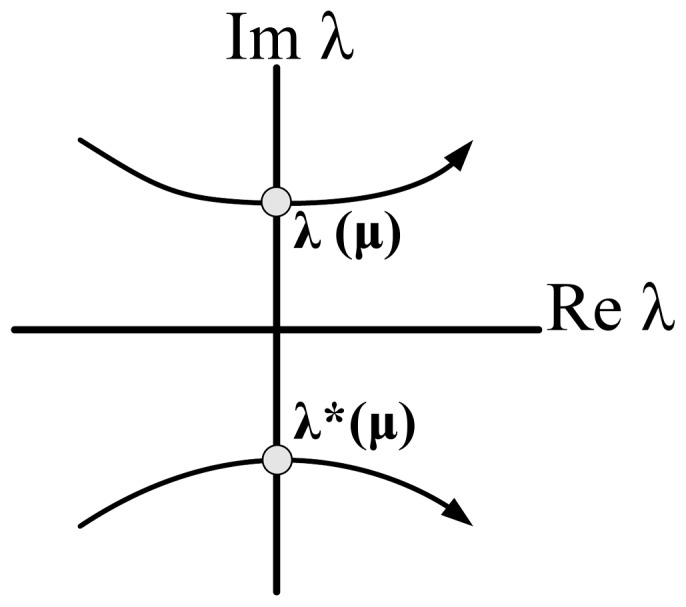
Locus of system's eigenvalues during the “*birth*” of a limit cycle. 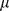
 is defined in [40] as a bifurcation parameter.

The use of the Andronov-Hopf bifurcation theorem is particularly useful to determine CytoMimetic circuits' frequencies of oscillations [37]. The formula 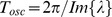, where 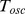 is the period of oscillations and 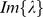 refers to the imaginary part of the eigenvalues calculated at the critical bifurcation point of a given system (see Figure 5), provides a means to estimate the period of oscillations as long as the bifurcation parameter is *“close”* to the critical bifurcation value. Further information on this can be found in [12], [40], [41].

For the models examined in this paper, the frequency of their oscillations could not be determined by the aforementioned method, since the systems' points of operation are far away from the critical bifurcation point. Consequently, we estimated the frequency of oscillations exclusively through the appropriate use of signal processing tools such as those found in Cadence and MATLAB^©^ software.

### Intracellular *Ca* ^2+^ oscillations model (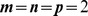 case)

The model of intracellular 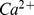 oscillations described by (1) is a two-dimensional model. Since two prototype differential equations are targeted, two electrical differential equations must be employed. Based on the analysis provided in section 5 the following steps have been followed:

- The time-varying concentration of cytosolic 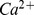 (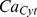) denoted by 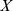 in (1) has been chosen to be implemented by means of the output current 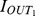 of the 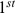 BC, which bears the subscript 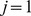 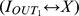.
- The time-varying concentration of 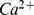 in the 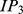-insensitive pool (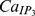) denoted by 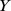 in (1) is implemented by means of the output current 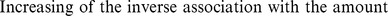 of the 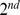 BC, which bears the subscript 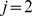 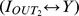.
- We have mapped each parameter and variable of the chemical model onto a current in the electrical equivalent one. Although such an approach might seem counterintuitive, especially in the case where the chemical value 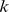 is characterised by units of 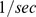, the rather flexible nature of the NBCF helps us overcome this problem. As illustrated in (18), the dimensionless parameter 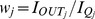 multiplied by the input/output BC currents 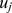 or 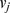 and by the 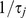 factor ensures that this product has dimensions of 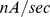, since the unit of the term 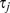 is 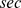. Indeed, the current 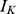 for example, which corresponds to the variable 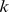 of the biological model is divided by 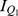 and multiplied by the 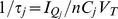 factor, which has units of 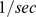 (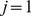 in this case).
- The correspondence between biological concentration and electrical current is 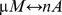.

Based on the above, we can start forming the electrical equivalent using only the first two terms of (18):
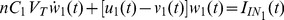
(21)

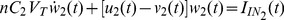
(22)According to (16) and (17), (21) and (22) can be re-expressed as:
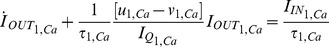
(23)

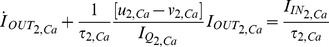
(24)For the realisation of the correct electrical equivalent equations, the appropriate 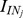, 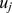 and 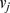 (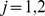) currents must be selected, as discussed in section 5. To elucidate the selection, (1) is re-written in a form that resembles (23) and (24). According to [1] and [19], in the case where 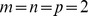, the time constant 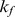 is zero. Furthermore, the parameter 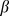 present in (1) has been substituted by 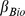, to distinguish it from the electrical 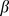. Thus, from (1) we have:
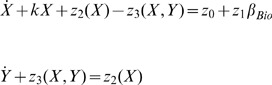
or
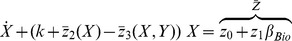
(25)

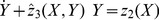
(26)where now
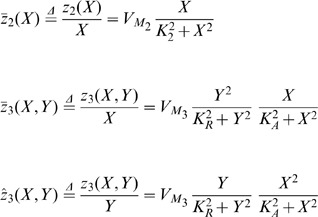
By comparing (25) to (23) and (26) to (24), we set the following 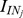, 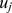 and 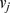 (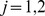) currents for 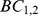, in order to map the biological parameters onto electrical ones:

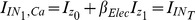
(27a)

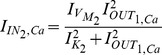
(27b)

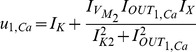
(27c)

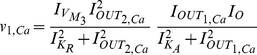
(27d)

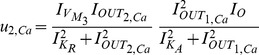
(27e)

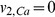
(27f)

where the 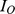 and 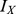 factors correspond to biasing currents employed by the squarers' and multipliers' blocks used to implement the appropriate mathematical operations (see Figures 3 and 4).

After the above treatment, substituting (27) into (23) and (24) yields:
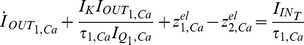
(28)

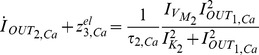
(29)where
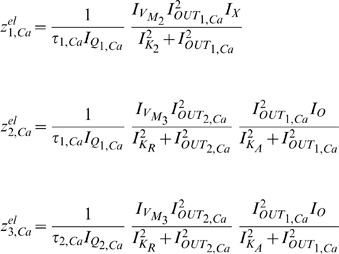

Table 1 summarises both chemical and electrical equations in a way that highlights the analogies between them. Unit consistency is preserved in (25), (26), (28) and (29) with the units of (25) and (26) corresponding to 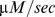 and the units of (28) and (29) to 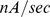 in a complete analogy.

**Table 1:** Chemical And Electrical Equations Of The Intracellular 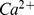 Oscillations Model (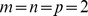) Case, Codified By (1), (28) 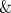 (29).

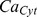	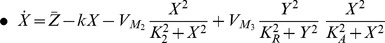	**Chemical Equation**
	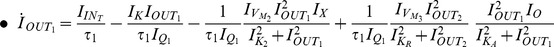	**Electrical Equation**
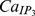	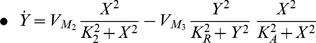	**Chemical Equation**
	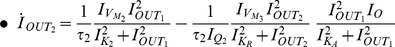	**Electrical Equation**

### Genetic regulatory networks model (two-dimensional case)

For the two dimensional case of the genetic regulatory networks model, the following steps have been followed:

- The time-varying behaviour of protein's 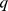 concentration is implemented by means of the output current 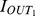 of the 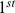 BC which bears the subscript 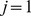 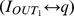.
- We have selected to implement the time-varying behaviour of protein's 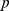 concentration by means of the output current 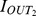 of the 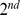 BC which bears the subscript 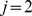 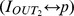.
- Each parameter and variable of the chemical model is mapped onto a current in the electrical equivalent one.
- The correspondence between the units of the prototype and electrical system is 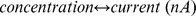.
- In the electrical model, the equivalent of the time scaling factor 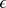 of the biological model (see (2)) has been implemented by means of a “*gain*” current termed 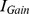, analogous to the value of 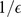 and by setting the values of the currents 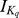 and 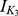 analogous to the values of 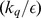 and 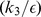, respectively.

The exact same procedure as before is adopted for the realisation of the electrical equations of this model from the prototype ones presented in (2). Starting once again from the general form of the NBCF in (18) we end up with the following two-dimensional electrical expressions:
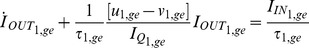
(30)

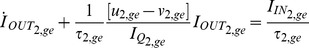
(31)By bringing the prototype equations of (2) into a form similar to (30) and (31), we can make the selection of the input and output currents of the two BCs more apparent:
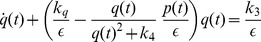
(32)

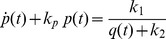
(33)A direct comparison of (30) with (32) and (31) with (33) helps us determine the following 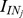, 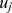 and 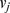 (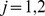) currents for 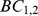, to achieve mathematical mapping of the biological terms onto the electrical ones:

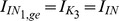
(34a)

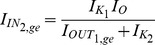
(34b)

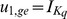
(34c)

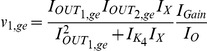
(34d)

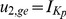
(34e)

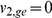
(34f)

where the 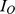 and 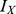 factors correspond to squarers' and multipliers' biasing currents.

Based on the above analysis and (34), the relations (30) and (31) are transformed as follows:
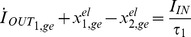
(35)

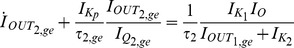
(36)where
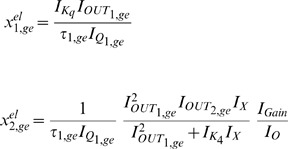

Table 2 summarises the prototype and electrical equations for the gene-protein regulation model.

**Table 2:** Chemical And Electrical Equations Of The Gene-Protein System Model, Codified By (2), (35) 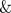 (36).

	Chemical Equations	Electrical Equations
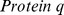	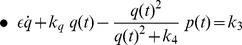	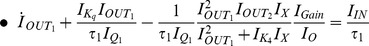
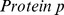	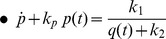	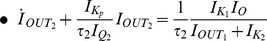

### Full circuit schematics

**Figure 6:**
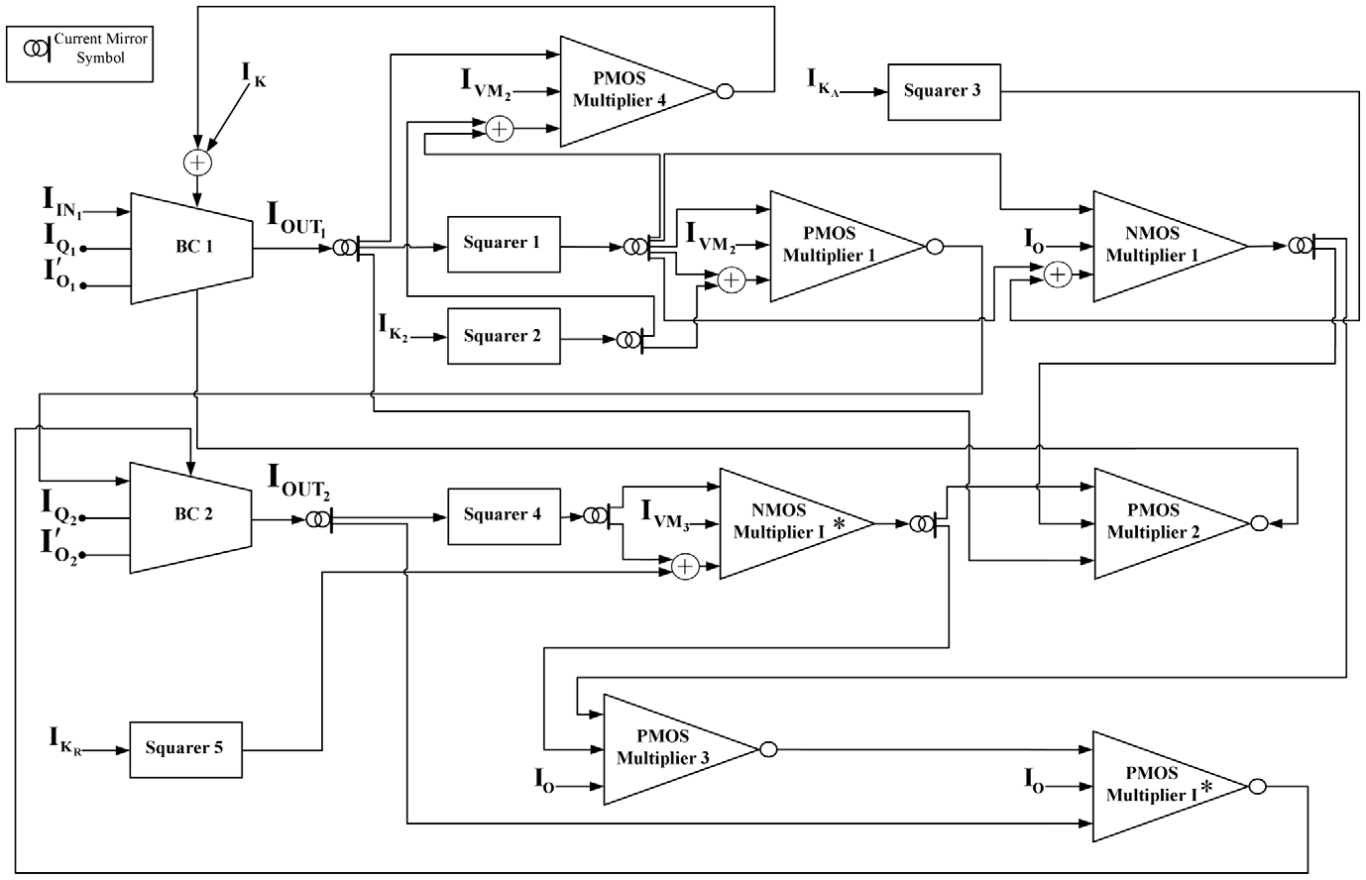
A block representation of the total circuit implementing intracellular *Ca* ^2+^ oscillations for the case with Hill coefficients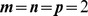 as codified by (28) 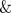 (29). Two TL blocks have been selected in a non-cascoded form to provide circuit stability for low power supply.

**Figure 7:**
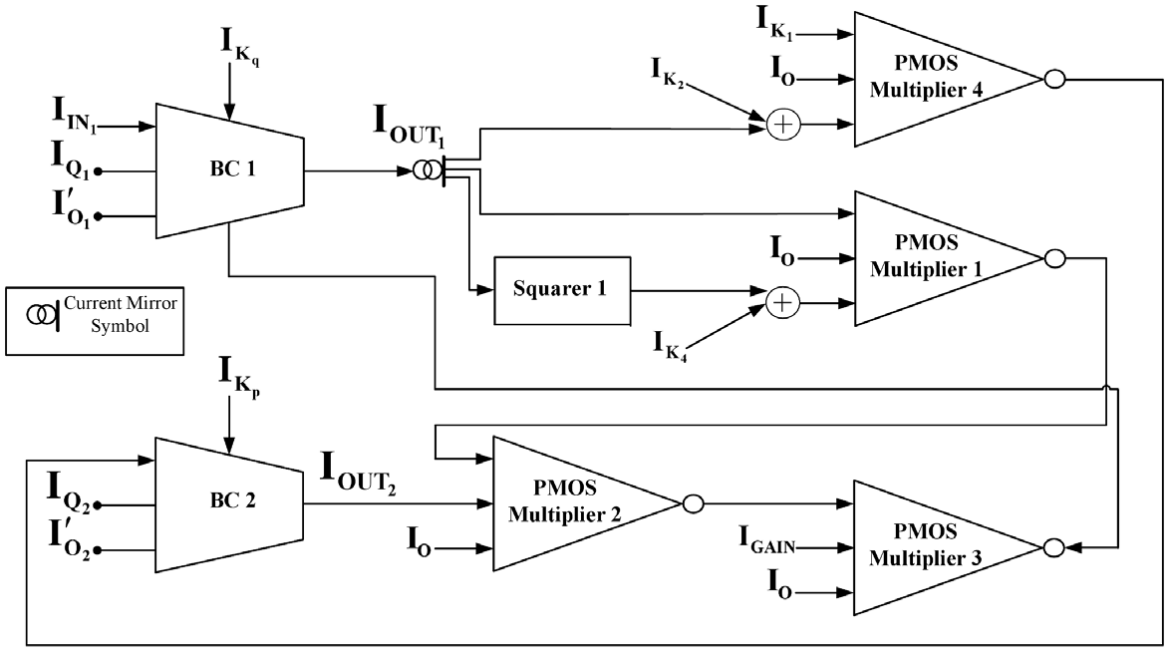
A block representation of the total circuit implementing the two-dimensional gene-protein regulation model as codified by (35) 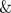
 **(36).**

Exploiting the symbolic representation of the basic TL blocks introduced in section 5, schematic diagrams for the two different biological models are presented in Figures 6 and 7. Through these diagrams one can understand how the equations in Tables 1 and 2 have been formed. For example, from Figure 7 one can track the formation of the electrical equation for protein q, shown in Table 2.

Starting from the general form of the 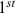 ODE of the system that is shown in (30) and is physically implemented by the 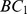 block, the input/output currents of the block need to be formed. Based on the analogy between biological and electrical model, from (32) it can be found that for the 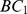 block's input current a constant current source of value 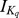 will be required. On the other hand, the output current 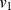, is clearly a combination of the output currents of 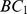 and 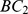, 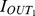 and 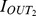. The PMOS multiplier 1 block combines 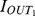 with its squared value and their product is subsequently combined with 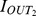 through the PMOS multiplier 2 block. The total product returns to the 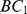 block as output current 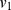 via the PMOS multiplier 3, where it is multiplied by the value of the current 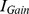. In an exact similar way the input and output current of all the other BC blocks of both electrical equivalent systems are formed.

## Mathematical Analysis of the Biological and Electrical Models

The characteristics of the oscillatory behaviour of both prototype and electrical models are determined by their Jacobian matrixes and eigenvalues. In the following paragraphs, the mathematical properties of the biochemical models and their electrical equivalents are analysed using the aforementioned linearised mathematical tools. The two models studied are the ones of section 6. At this point, it would be useful to add that the remaining models (see section 2) have also been investigated in a similar way and yield similar results.

### Intracellular calcium oscillations model (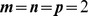 case)

#### Biochemical model

**Figure 8:**
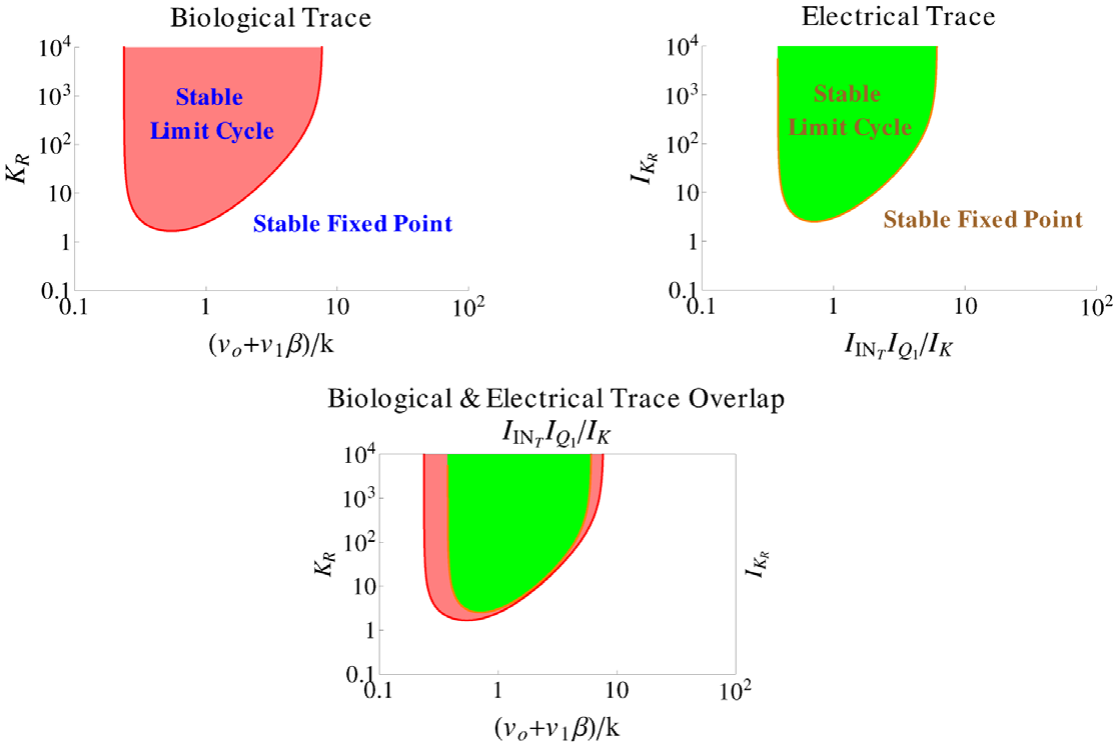
Regions of oscillations (shaded parts) for both prototype and electrical intracellular *Ca* ^2+^ oscillations systems, based on their traces illustrated in Table 3. A relation between 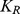 and 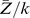 and 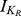 and 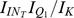 has been plotted in complete analogy to [1]. The values been used for the calculation of both areas are shown in Tables 5 and 9.

By setting the derivatives of the model in (25) and (26) equal to zero and solving for 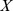 and 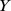, the fixed points 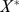 and 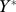 of the system can be calculated:
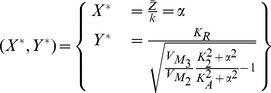
The Jacobian matrix of the system is:
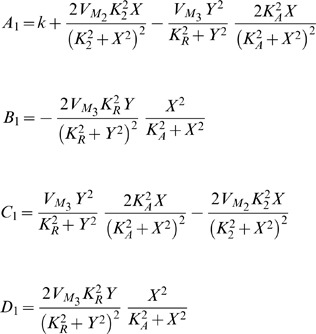
where
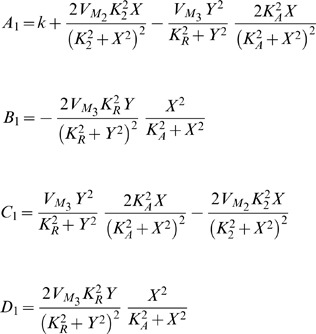
The following conditions are necessary for the generation of sustained oscillations; the imaginary eigenvalues of the system 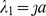 and 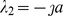 must satisfy the following: (a) 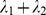 = 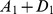 = 0 and (b) 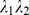 = 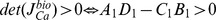. Moreover, from the above Jacobian matrix a pool of values, within which the system exhibits sustained oscillations, can be determined. In order to define this region of oscillations, the trace of the Jacobian matrix (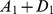) is set equal to zero after verifying that the determinant is positive for these values. Table 3 summarises the outcome of this calculation and produces the left shaded region of oscillations illustrated in Figure 8, which is similar to the one presented in [1].

**Table 3:** Regions Of Oscillations For Intracellular 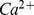 Biological Model And Its Electrical Equivalent.

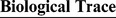
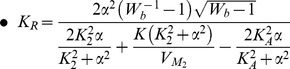
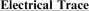
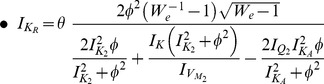
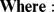
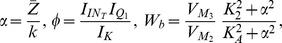
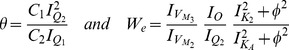

#### Electrical equivalent model

Setting both derivatives of the electrical equivalent system equal to zero and solving for 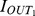 and 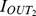, the following fixed points 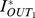 and 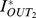 can be calculated:
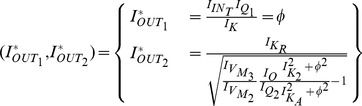
The similarity between the electrical and biological fixed points is straightforward. In a similar way as before, the Jacobian matrix of the system can be computed:
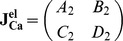
where
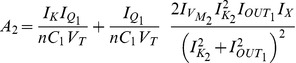


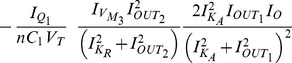


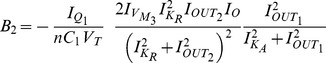


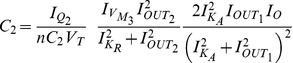


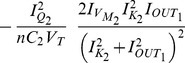


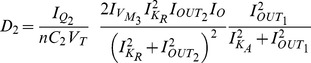
For the generation of sustained oscillations in the electrical equivalent system, the same conditions as in the biochemical model case should apply for the electrical eigenvalues. The equation that defines the electrical region of oscillations has been generated by setting the electrical trace (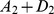) equal to zero and is also codified in Table 3. The region of oscillations of the electrical equivalent model corresponds to the right shaded area presented in Figure 8.

### Gene regulatory networks model (two-dimensional case)

#### Biochemical model

Following the analytical steps detailed in [24], the fixed points 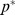 and 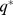 of the mathematical model (32) and (33) are calculated as follows for the parameter values reported in [24]:
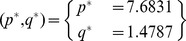
The Jacobian matrix becomes:
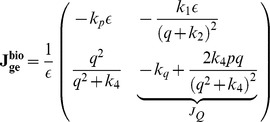
According to [24], it is the sign of 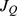 in the Jacobian matrix which defines whether an oscillation occurs or not. Based on the proof presented in [24], the system exhibits oscillatory behaviour when the term 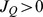, while when 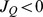 the system demonstrates steady behaviour.

#### Electrical equivalent model

The fixed points 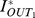 and 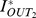 of the gene-protein electrical circuit (35) and (36) become:
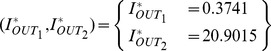
The Jacobian matrix of the electrical equivalent is defined as follows:
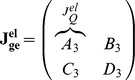
where
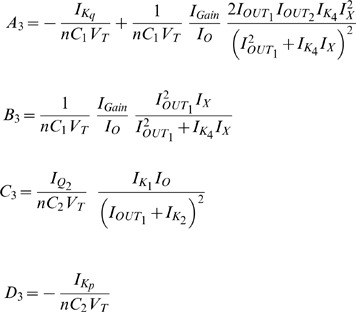



Following the analysis in [24], when 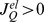 the electrical equivalent circuit oscillates, while it remains steady for 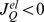. This can be verified by using the electrical values presented in the following sections for this type of circuit.

## Simulation Results

This section aims at demonstrating the correspondence between the dynamical behaviours generated by simulating both the biochemical/prototype and the electrical models. The software used for the simulation of the aforementioned circuits is Cadence Design Framework (CDF) version 5.1.41, using the process parameters of the commercially available *AMS 0.35* 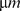 *- MM/2P4M c35b4 CMOS* technology. MATLAB^©^ and Cadence results have been obtained for certain biological and electrical parameters. The biological parameters' values have been acquired from literature, while the electrical parameters have been calculated from the scaled relation between the two systems. The scaling factors, aspect ratios and capacitance values presented in Tables 4, 5, 6, 7, and 8 and Table 9, respectively, are not unique. Further explanation regarding the values of these quantities will be provided in the following paragraphs.

**Table 4:** Biological And Electrical Values For The 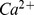 Oscillations Model (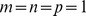 Case).

Biological Values		Electrical Values (Scaling Factor 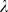 : 50%)
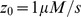	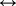	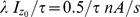
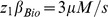	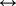	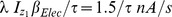
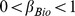	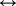	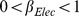
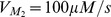	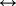	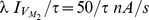
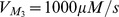	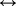	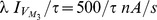
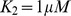	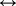	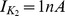
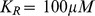	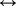	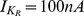
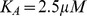	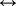	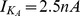
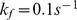	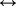	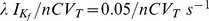
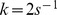	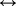	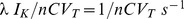

**Table 5:** Biological And Electrical Values For The 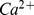 Oscillations Model (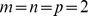 Case).

Biological Values		Electrical Values (Scaling Factor 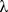 : 10%)
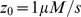	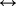	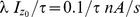
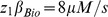	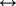	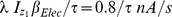
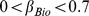	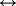	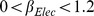
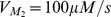	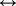	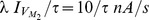
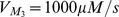	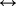	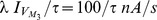
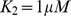	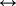	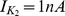
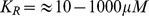	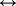	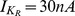
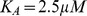	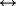	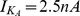
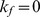	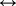	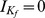
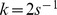	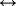	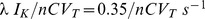

**Table 6:** Biological And Electrical Values For The 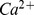 Oscillations Model (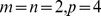 Case).

Biological Values		Electrical Values (Scaling Factor 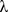 : 25%)
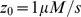	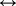	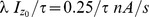
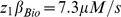	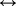	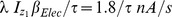
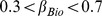	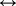	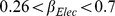
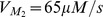	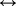	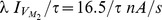
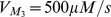	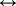	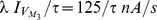
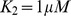	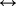	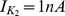
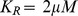	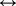	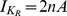
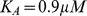	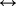	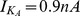
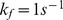	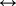	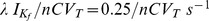
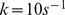	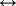	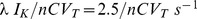

**Table 7:** Biological And Electrical Values For The Gene-Protein Regulatory Model (2D - Case) for 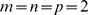.

Biological Values		Electrical Values (Scaling Factor 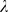 : 50%)
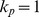	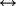	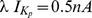
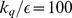	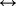	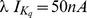
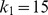	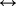	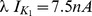
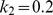	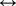	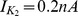
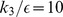	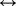	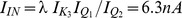
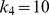	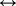	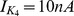
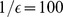	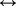	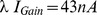

**Table 8:** Biological And Electrical Values For The Gene-Protein Regulatory Model (3D - Case) for 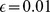.

Biological Values		Electrical Values
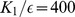	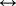	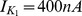
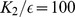	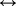	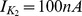
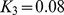	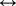	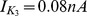
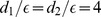	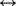	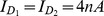
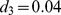	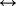	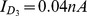
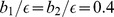	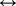	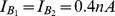
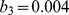	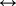	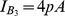
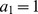	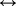	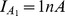
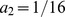	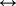	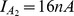
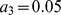	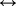	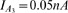

**Table 9:** Electrical Properties Of Log-Domain Intracellular *Ca* ^2+^ Oscillations & Gene-Protein Regulatory Circuits.

**Type Of Log-Domain Circuit**	*Ca^2+^(m = p = 1)*	*Ca* ^2+^(*m* = *n* = *p* = 2)	*Ca* ^2+^(*m* = *n* = *2, p* = 4)
Power Supply (Volts)	4	2	2.5
*I_Q_1__* (*nA*)	0.8	0.95	0.95
*I_Q_2__* (*nA*)	0.8	0.95	0.95
*I_O_* = *I_X_* (*nA*)	1	1	1
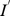 _O_ (*nA*)	5	1	0.1
Capacitances (*pF*)	*C_1_ = C_2_* = 190	*C_1_ = C_2_* = 200	*C_1_ = C_2_* = 250
W/L ratio of PMOS and NMOS Devices (*μm* / *μm*)	200/1.5	30/9 and 10/2	28/8 and 8/1
Static Power Consumption (*μW*)	12.61	6.49	1.53
Number of devices (including current mirrors)	205	247	252
Chip Area (On Chip Caps/Off Chip Caps) (Estimate - in *mm* ^2^)	0.533/0.0718	0.537/0.079	0.661/0.0911

### Log-domain intracellular *Ca* ^2+^ oscillations circuits

**Figure 9:**
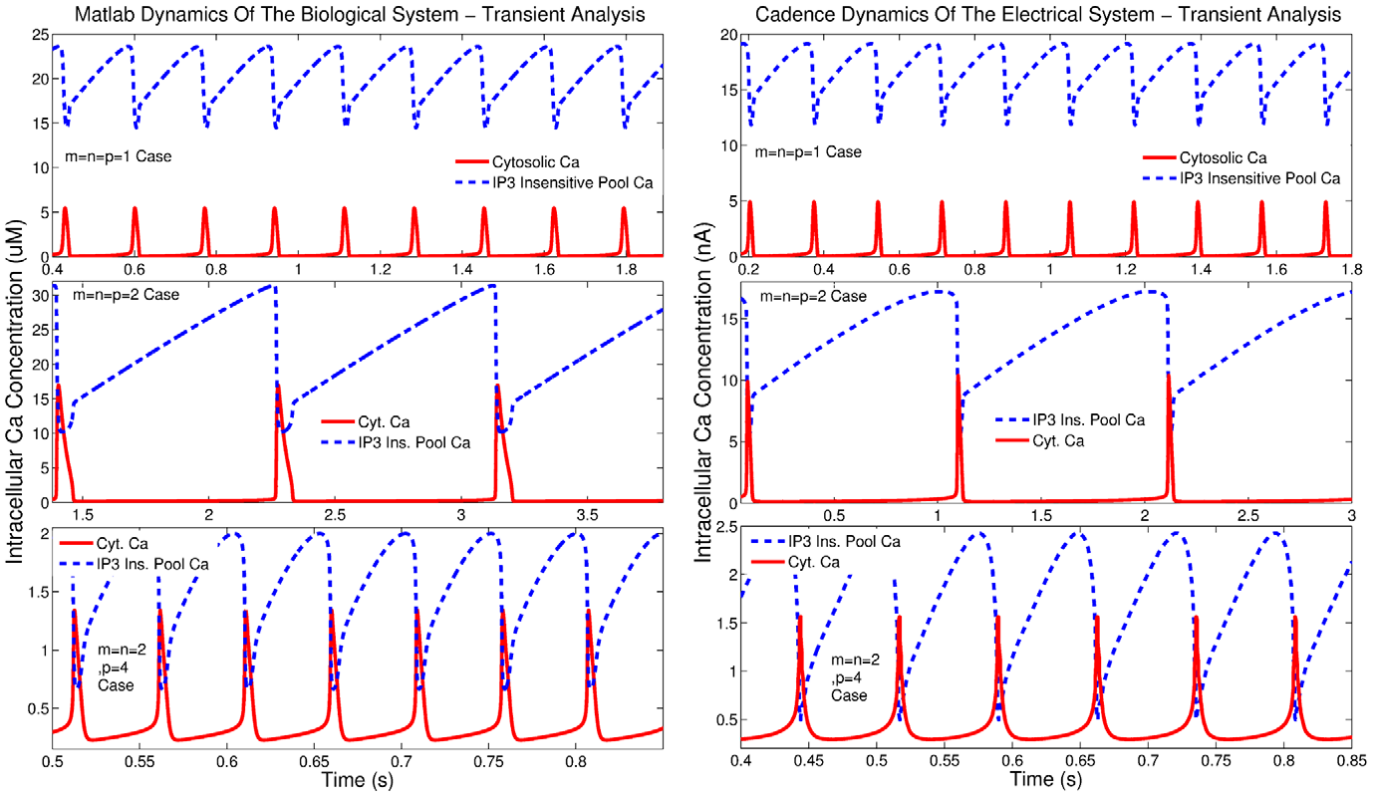
Comparison of transient analysis results generated by MATLAB© and Cadence simulations for the Log-Domain intracellular *Ca* ^2+^ oscillations circuits.

**Figure 10:**
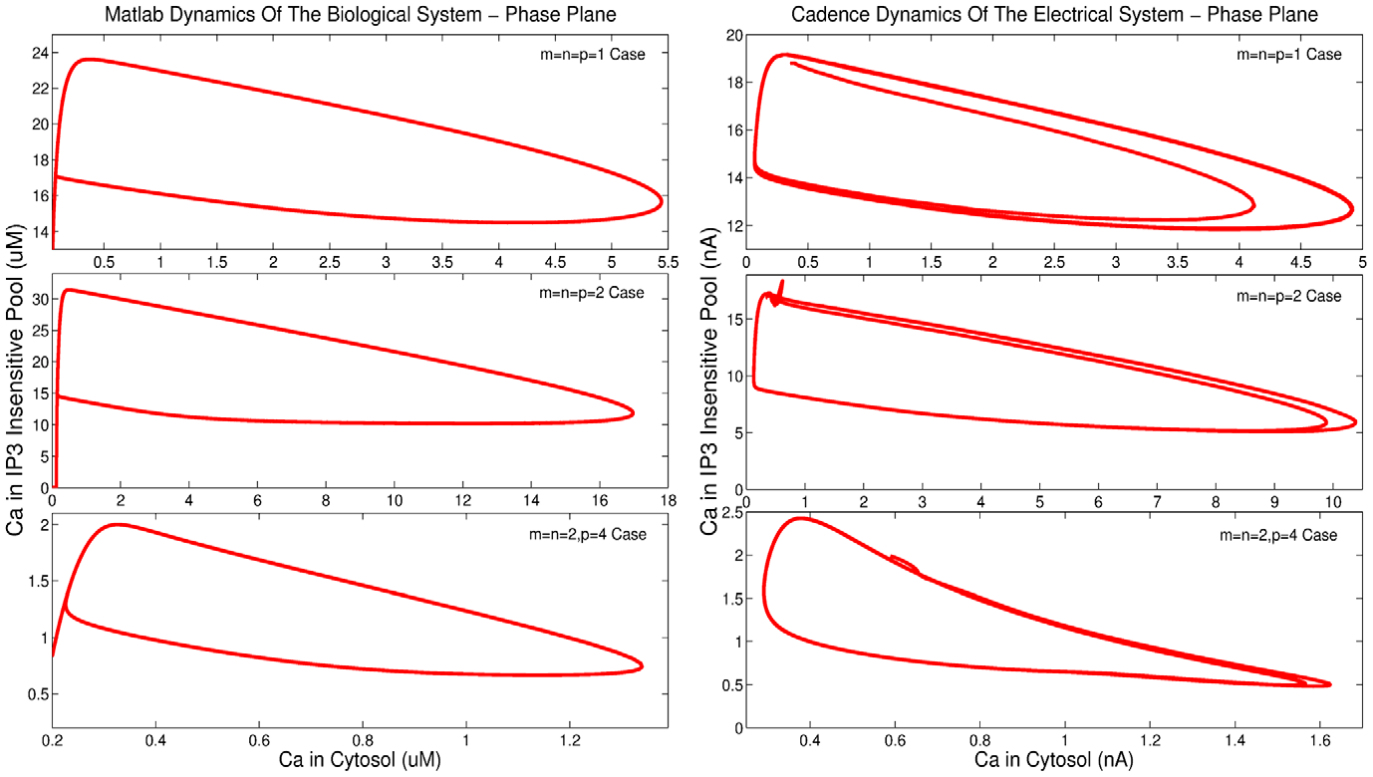
Comparison of phase plane analysis results generated by MATLAB© and Cadence simulations for the Log-Domain intracellular *Ca* ^2+^ oscillations circuits.

The proposed circuits can operate with different values of the aforementioned quantities and produce similar dynamical behaviours as the ones illustrated in Figures 9 and 10. The reported values are an indicative example leading to small chip area and low power consumption, without being the only ones with these characteristics. Scaling of the electrical current values was required, in order to ensure compliance with the weak-inversion conformities. It has been achieved by multiplying the values of the constant currents existing in the numerators of the electrical ODE, such as 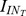, 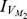, 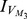 and 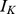 (see Table 1) by a scaling factor. By doing so, the electrical circuit's time parameter 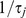, with 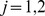 is multiplied by this scaling factor leading to a time scaled final electrical system. The time axis of the biological simulation figures presented in Figure 9 needed to be normalised with respect to the electrical systems' time axis for the sake of comparison. It has been achieved by multiplying the biological ODEs (see (1)) by the constant 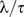, where 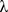 is the scaling factor and 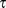 the time parameter of each electrical system.

#### 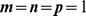 case simulation parameters

The first case of the intracellular 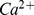 model demonstrates that the mechanisms of pumping, release and activation can be described by intrinsic Michaelian processes. Based on [1] and [19], the various values of the biological and electrical model parameters are presented in Table 4. The electrical equivalent equation for this system is not presented due to lack of space, however, it has been left to the interested reader to verify the similarity between the aforementioned equations and the ones presented in Table 1.

As can be seen from Table 4, a scaling factor of 0.5 has been applied to certain electrical quantities, forming a scaled electrical equivalent model and without affecting the validity of the mathematical model. Since the initial parameter values of this biochemical model were relatively high for weak-inversion region current values, the introduction of this scaling factor facilitates the compliance of the proposed circuit with the logarithmic conformities.

Both MATLAB^©^ and Cadence results presented in Figures 9 and 10, for this case of 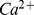 oscillations, have been generated for 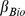 = 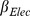 = 0.01. The remaining electrical parameters, such as the values of the shifting currents 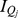, 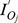, the values of the biasing currents 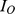 and 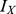, aspect ratios and capacitances (see Figures 2, 3, and 4) are reported in Table 9, which summarises the electrical parameters of the circuits simulated and commented up in the next section.

**Figure 11:**
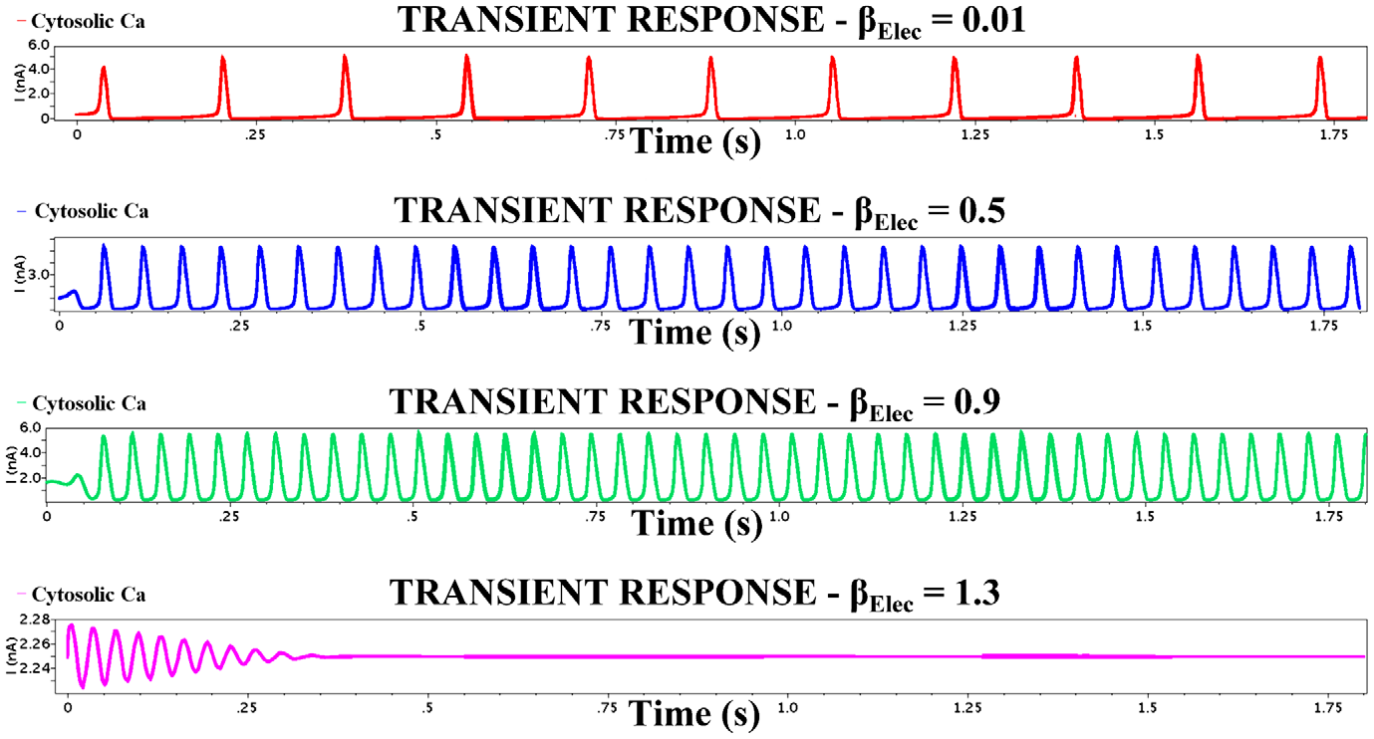
Transient analysis of the 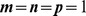 intracellular *Ca* ^2+^ circuit simulated for the values shown in Table 4 and for four different 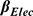 values. The electrical parameters are listed in Table 9. The figure illustrates the temporal behaviour of cytosolic 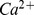 as the value of the parameter 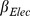 increases. Increasing the value of 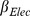, one can observe that the attractor of the system changes from an asymptotically stable limit cycle to an asymptotically stable fixed point. Damped oscillations are generated when the system “*crosses*” the bifurcation point of the system, which takes place when 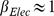.

The aforementioned simulation results demonstrate good qualitative agreement with each other. The signature of the electrical nonlinear system, i.e. the system's phase plane, shows good agreement with the biological one generated by MATLAB^©^. Moreover, simulation results have been performed for various capacitance values to investigate circuit's robustness. The vast majority demonstrated good agreement with MATLAB^©^ simulations for the values presented in Table 4 suggesting that the chip area could decrease without affecting the targeted dynamics significantly. Finally, Figure 11 demonstrates the actual circuit's behaviour as the parameter 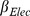 increases. In practice, the electrical system is migrating towards its bifurcation point, which leads to the transfer from periodic to damped system oscillations.

#### 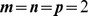 case simulation parameters

The second case of the intracellular 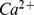 oscillations model is characterised by a Hill coefficient of 2 and - in principle - represents a less mild nonlinear system, compared to the previous case. The values of the biological model are reported in [1], [13], [18]–[22] and similarly to the previous case, a scaling factor of 0.1 has been introduced for the values of the electrical equivalent model. The remaining values for both models are presented in Table 5. The simulation results shown in Figures 9 and 10, for this case, correspond to 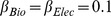 and 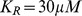. The rest of the electrical model parameters regarding shifting and biasing currents, aspect ratios and capacitances are being codified in the collective Table 9. It should be mentioned that although the value of 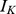 should be equal to 0.2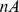 based on the proposed scaling, it has been found that a value of 0.35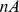 leads to slightly better transients and Monte Carlo Analysis results. “*Calibrating*” this current value served only presentation purposes aimed at highlighting the resemblance between a real, electrical circuits response and the one produced in MATLAB©. As it will be discussed in section 9, minor deviations from the ideal prototype system are a *“feature”* of this proposed class of circuits. In this case as well, transient and phase plane analysis demonstrates that the two systems are adequately close. However, differences exist at the boundaries of the regions of oscillations for these systems, as illustrated in Figure 8.

#### 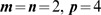 case simulation parameters

**Figure 12:**
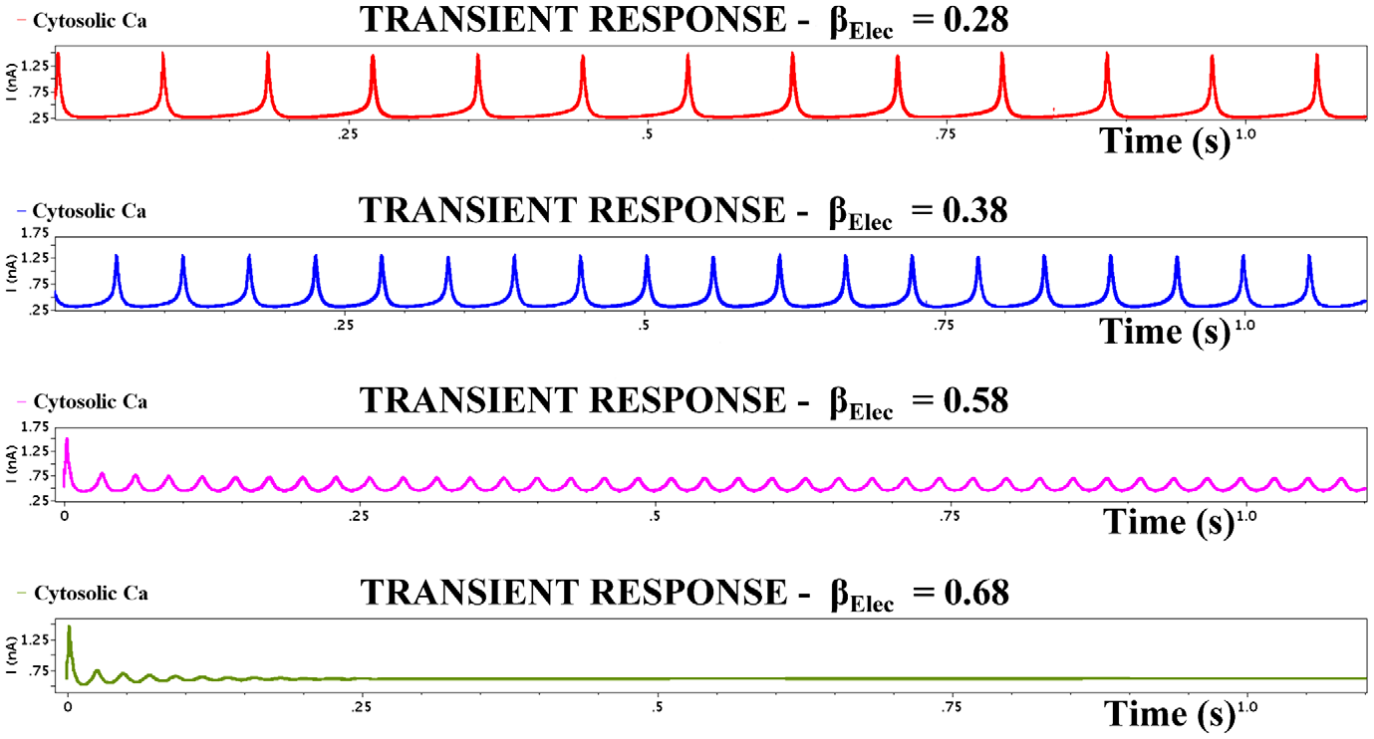
Transient analysis of the 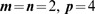 intracellular *Ca* ^2+^ circuit simulated for the values shown in Table 6 and for four different 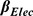 values. The electrical parameters are listed in Table 9. The figure illustrates the transition of the electric system from asymptotically stable limit cycles to asymptotically stable fixed points. Damped oscillations are again generated after the system's bifurcation point, which corresponds to 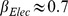. The simulated results exhibit satisfying resemblance with the simulation graphs presented in [1].

The third case of the intracellular 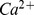 oscillations model is the one with the highest-order of Hill coefficients equal to 4, leading inevitably to a stronger nonlinear behaviour, where small current value deviations can significantly alter the targeted dynamics. The selection of the biochemical parameter values can be found in [1], [13], [18]–[22] and as before the electrical parameters have been selected in a way that serves the successful circuit operation. Again, certain biochemical parameter values carried large values, thus, a scaling factor of 0.25 has been introduced as shown before. Table 6 summarises the correspondence between the values of the parameters of both models. The simulated results presented in Figures 9 and 10, for this case, have been obtained for 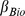 = 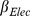 = 0.35. Shifting and biasing currents, aspect ratios and capacitances, corresponding to the rest of the parameters of the electrical equivalent model are again listed in Table 9. As in the 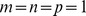 case, the migration of the electrical system towards damped oscillatory behaviour is illustrated in Figure 12 by increasing the 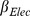 value. This behaviour complies with the behaviour of the prototype system as presented explicitly in [1].

**Figure 13:**
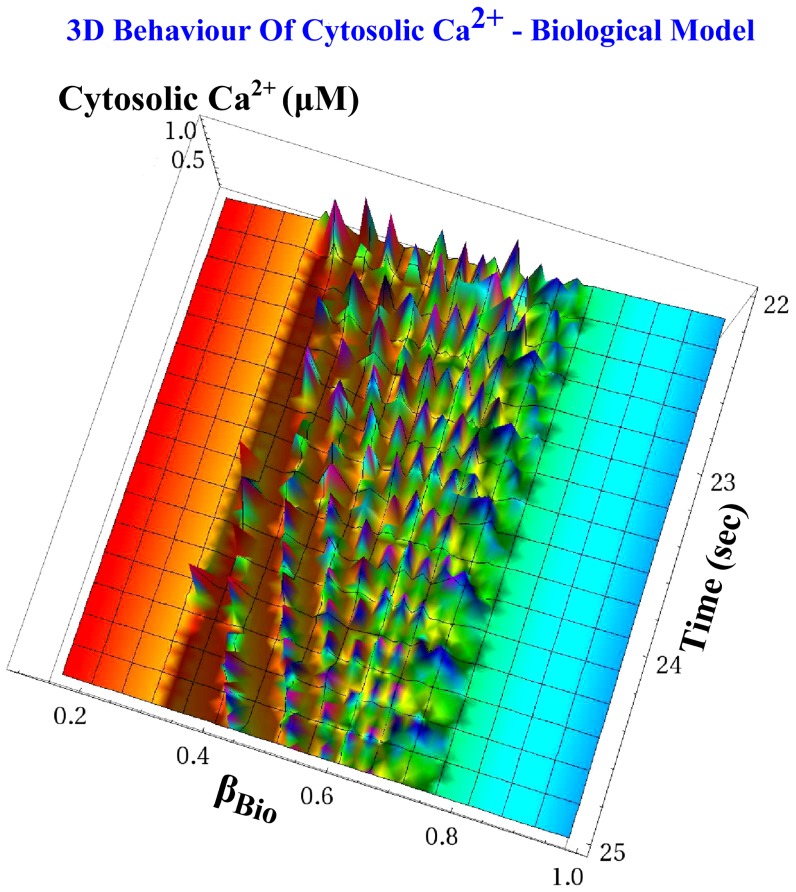
Three-dimensional representation of the cytosolic *Ca* ^2+^ oscillations based on the ideal biological model equations (1) for the 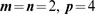 case. Using parametric sweep analysis with respect to the 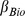 parameter, the birth and the decay of the cytosolic 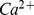 oscillations is presented. As expected, oscillations occurred only when 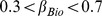.

**Figure 14:**
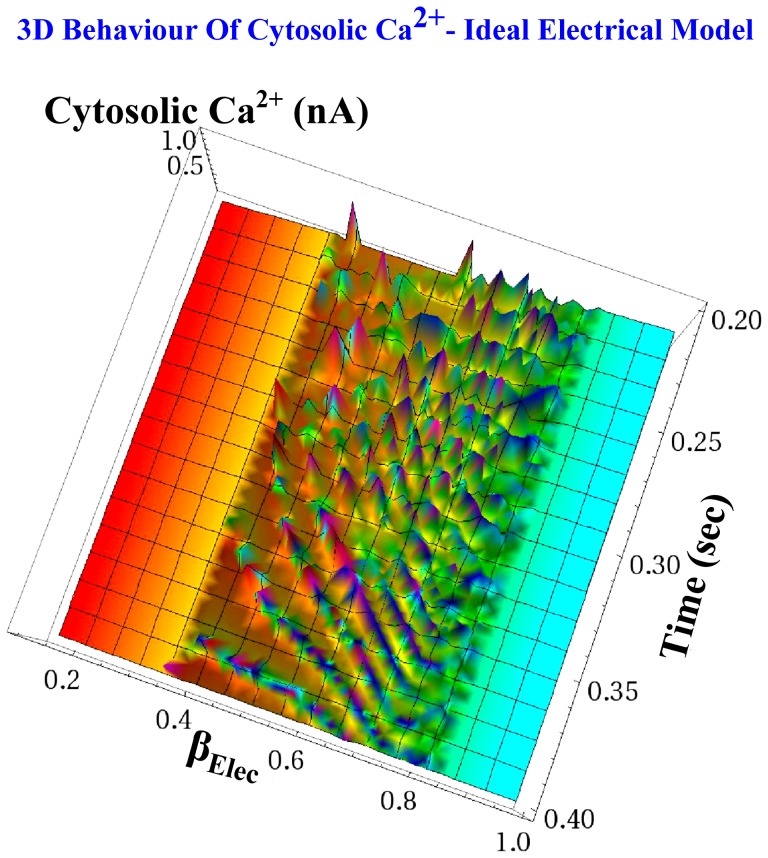
Three-dimensional representation of the cytosolic *Ca* ^2+^ oscillations based on the ideal electrical model equations for the 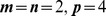 case. Using parametric sweep analysis with respect to the 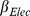 parameter, the birth and the decay of the 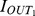 current oscillations that corresponds to cytosolic 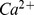 oscillations is presented. As expected, oscillations occurred only when 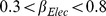. However, as Figure 12 illustrates, the real circuit implementing this category of 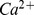 oscillations presented slight deviations regarding the boundaries where oscillations occurred, due to the non-ideal behaviour of the circuit's components.

This electrical equivalent circuit is the one with the less “*strikingly similar*” simulation results in the set we considered. The non-ideal exponential behaviour of certain devices combined with the strong nonlinearity of the model leads to noticeable deviations from the expected time traces and operating frequency, when the circuit's values are not identical to the corresponding biological ones. Finally, two three-dimensional graphs are shown, in order to demonstrate the behaviour of cytosolic 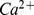 as 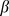 value increases. Figure 13 illustrates the behaviour of the cytosolic 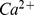 spikes based on the biological model, as shown in (25). As 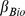 increases, the density of the spikes increases in total agreement with Figure 12. On the other hand, Figure 14 presents the three-dimensional behaviour of the ideal electrical equivalent circuit that implements cytosolic 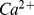 and is codified by (28). The similarities between the two figures are satisfying. Minor disagreement is observed for the value of 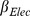 that defines the transition of the system from stable limit cycles to stable fixed points. For the biological system, it is clear from Figure 13 that this point occurs when 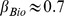, while for the ideal electrical one this point occurs when 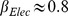.

### Log-domain gene-protein regulatory circuits

This class of mathematical models presents milder nonlinearities compared to the intracellular 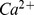 oscillation models.

#### Two dimensional model simulation parameters

The explicit mathematical analysis of this model takes place in [24] and the simulation results reported there have been collected using the set of values shown in Table 7. The units of the model are defined as “*concentration/time*” in [24]. The electrical equivalent model's parameter values are also listed in Table 7, scaled by a factor of 0.5. As in the 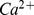 model case, several scaling factor values lead to similar dynamics.

**Figure 15:**
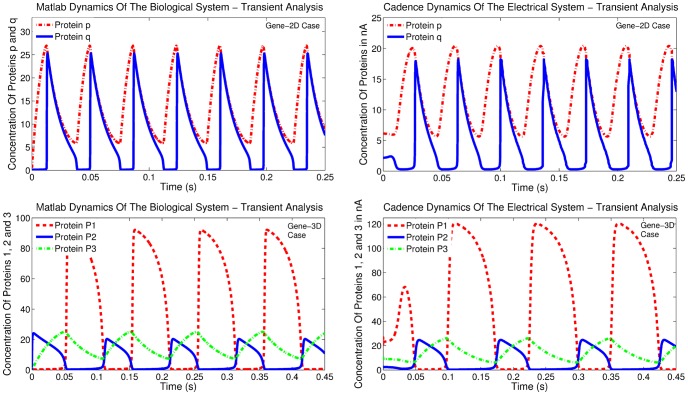
Comparison of transient analysis results generated by MATLAB© and Cadence simulations for the gene-protein regulatory circuits.

**Figure 16:**
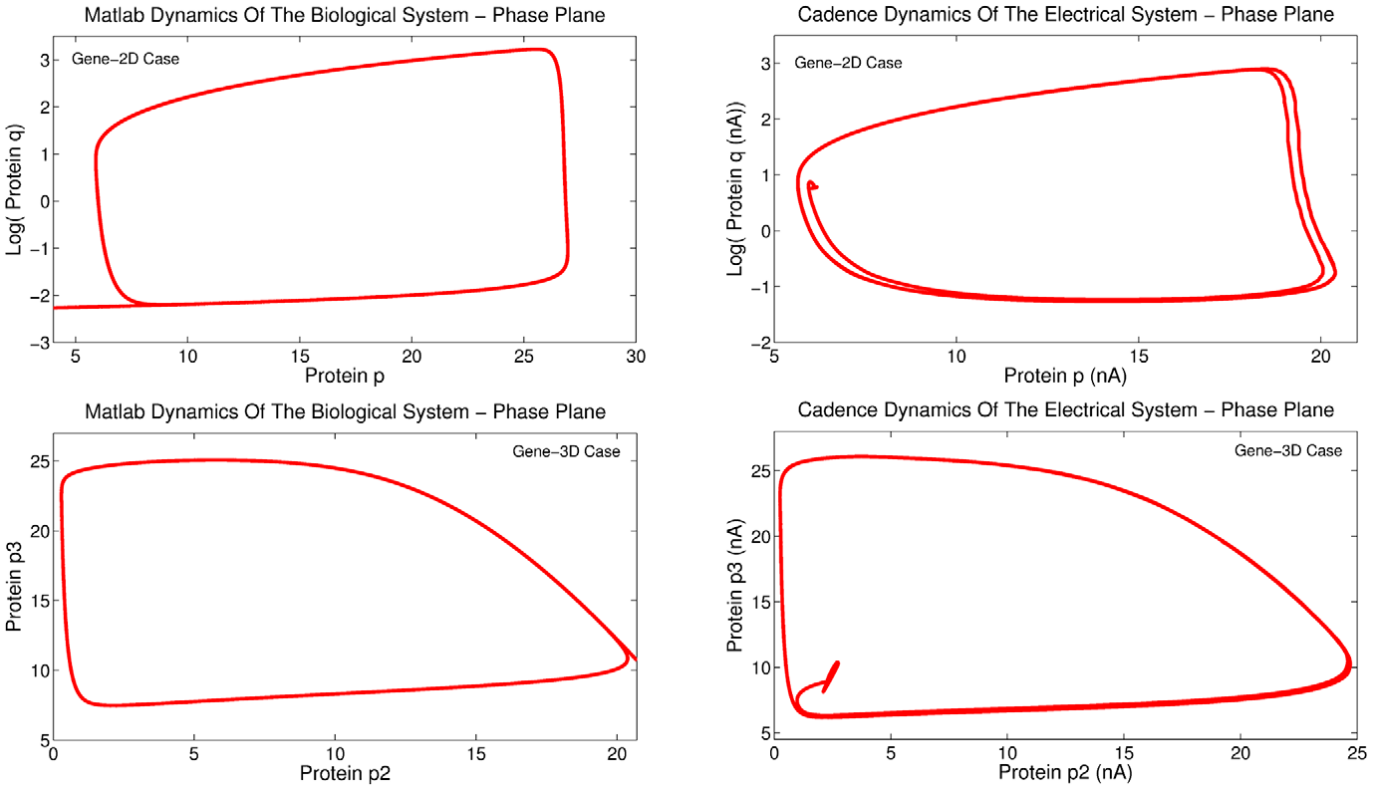
Comparison of phase plane analysis results generated by MATLAB© and Cadence simulations for the gene-protein regulatory circuits.

**Figure 17:**
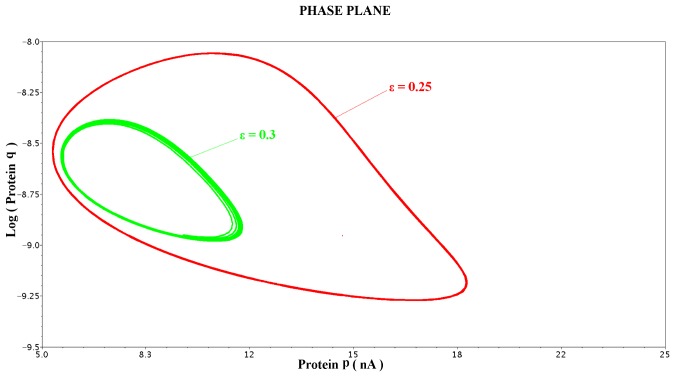
Phase plane analysis for the 2D gene-protein regulatory circuit with the 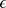 values set to 0.25 and 0.3. The presented results comply with a similar phase plane analysis presented in [24] for the same values of 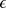.

The MATLAB^©^ transient and phase plane results illustrated in Figures 15 and 16 have been performed with the time scaling factor 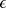 equals to 0.01. Cadence simulation results for 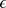 values of 0.25 and 0.3 are presented in phase plane form in Figure 17. The rest of the electrical parameters required for the implementation of the electrical equivalent circuit are again summarised in Table 9.

In the biological model the parameters 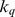, 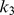 and the term 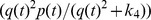 are divided by the time scaling factor 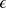, as discussed briefly in Section 6. Since in the electrical model, every parameter of the biological model has been represented by a current of analogous value, the multiplication of the terms 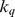 and 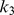 by 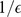 could be represented by specific currents 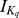 and 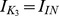 with values analogous to 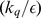 and 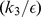, respectively. Consequently, the value 0.01 of the biological 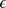 leads to electrical current values that are 100 times bigger than the original biological values. Moreover, the current 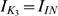 must be also multiplied by the factor 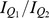 to ensure that the time constant parameter 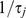 is similar for every electrical ODE of this electrical equivalent model, since in this circuit 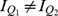.

Regarding the multiplication of the factor 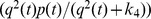 by 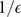 in the biological model, in the electrical equivalent model the multiplication can be achieved using two different techniques. The first involves the multiplication of the factor 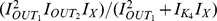 (see Table 2) by a gain current, which has the value of the biological 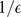. The second approach involves the use of a current mirror of ratio 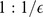. This ensures that the factor 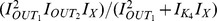 will acquire a value of 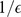 times larger than before. The first approach has been adopted for the simulations presented in Figures 15 and 16, while the second one for the phase plane results of Figure 17.

Finally, it is important to clarify that although the value of the current 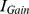 should have been equal to 50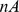 from a strictly mathematical point of view, it has been found that when 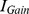 equals 43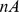 the circuits approximates better its ideal electrical response. This current value is translated into a biological time scaling factor of 0.0116, a value that is practically close to the theoretical value of 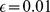. As already mentioned, “*calibration*” is not compulsory for this type of circuits, however, for presentation's sake we have decided to do so, in order to exhibit the potentials of the proposed circuits. Although this type of biological system has been realised via two different, transistor-level approaches, both of them exhibit good agreement with the theoretical transient and phase plane results.

#### Three dimensional model simulation parameters

The three dimensional case of the gene-protein regulation model is the only three dimensional system included in this paper. The reason that has led to its selection is twofold. The first relates with the fact that the noticeable wide range of its values (from a few 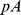 to hundreds of 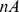) poses a challenging nonlinear model for testing both the validity and the flexibility of the NBCF. The second one aims at demonstrating the validity of the NBCF for higher order systems.

For this model, the authors in [24] have selected coefficient values that are presented in Table 8. In the same table the values of the electrical equivalent model parameters are tabulated. In this circuit case, there has been no scaling between the values of the original and the proposed electrical model. The time scaling factor 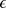 has been set at 0.01, as in the original paper. Since in the biological model the scaling factor 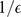 is multiplied only by terms that are constants, such as 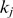, 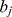 and 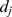, where 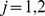, in the electrical equivalent model the currents 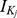, 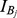 and 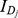 with 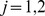 corresponding to the aforementioned biological parameters can bear values that are equivalent to 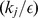, 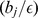 and 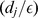, respectively, where 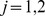. The rest of the electrical model parameters regarding shifting and biasing current values, device aspect ratios and capacitance values can be found in Table 9.

This only case of three dimensional model demonstrates good agreement with the theoretically expected behaviour as it can be observed from Figures 15 and 16. Despite the wide variety of the selected currents for the targeted dynamics implementation, the system behaves reliably, providing the desirable outputs. With regards to the small (4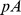) current value 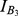, it is worth noting that it can be generated on-chip by means of ratiometric downscaling of a larger in value reference current.

## Robustness and Electrical Properties of CytoMimetic Circuits

**Figure 18:**
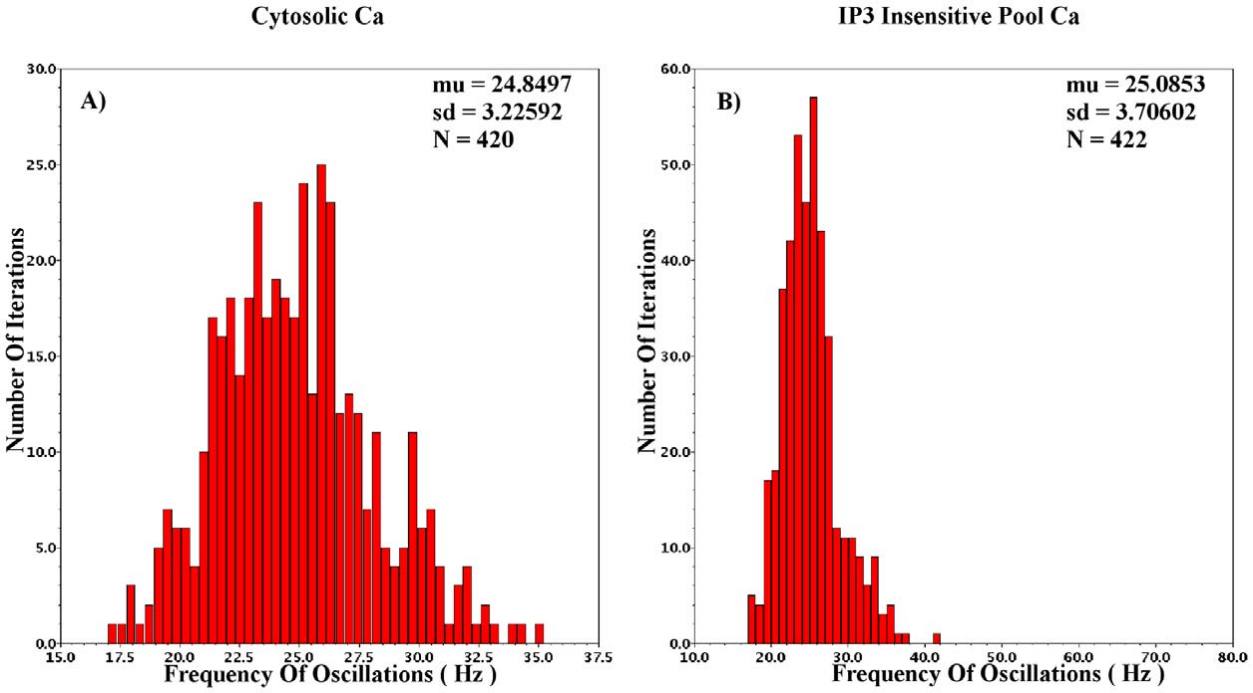
Monte Carlo analysis for the 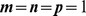 intracellular *Ca* ^2+^ Log-Domain circuit. 600 iterations have been performed and the percentage of iterations corresponding to successful oscillations was above 70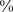.

**Figure 19:**
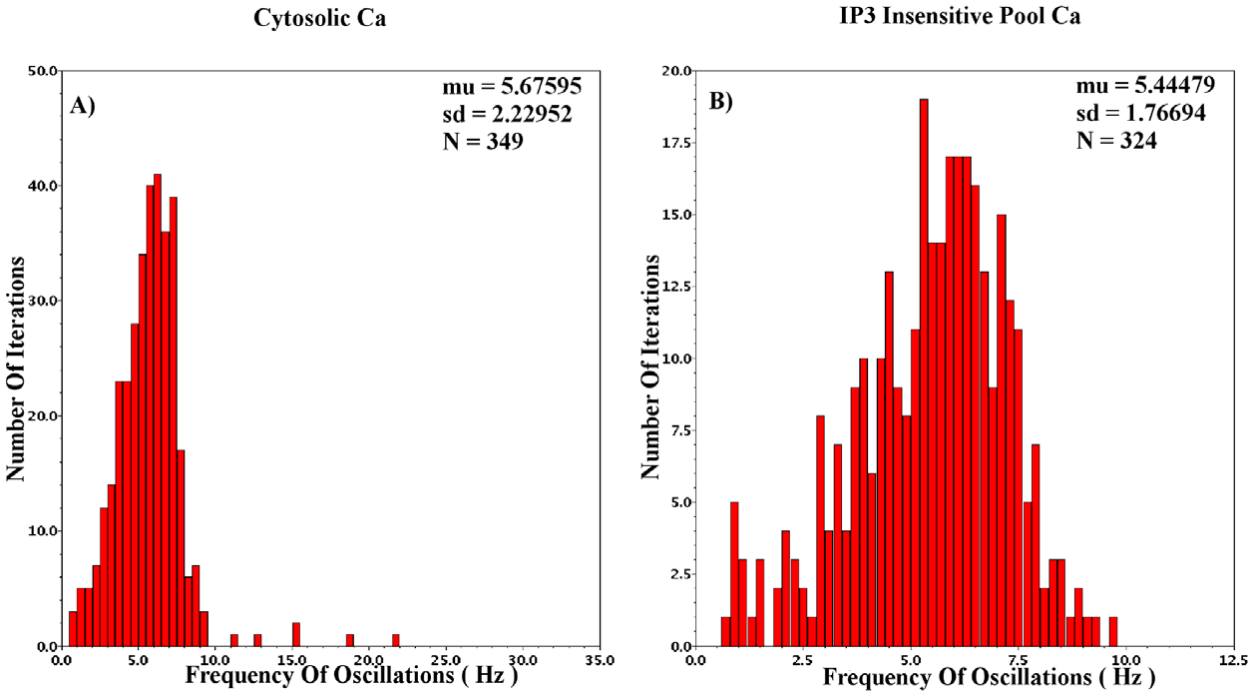
Monte Carlo analysis for 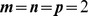 intracellular *Ca* ^2+^ Log-Domain circuit. From the 600 total iterations, more than 55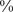 led to successful oscillations.

**Figure 20:**
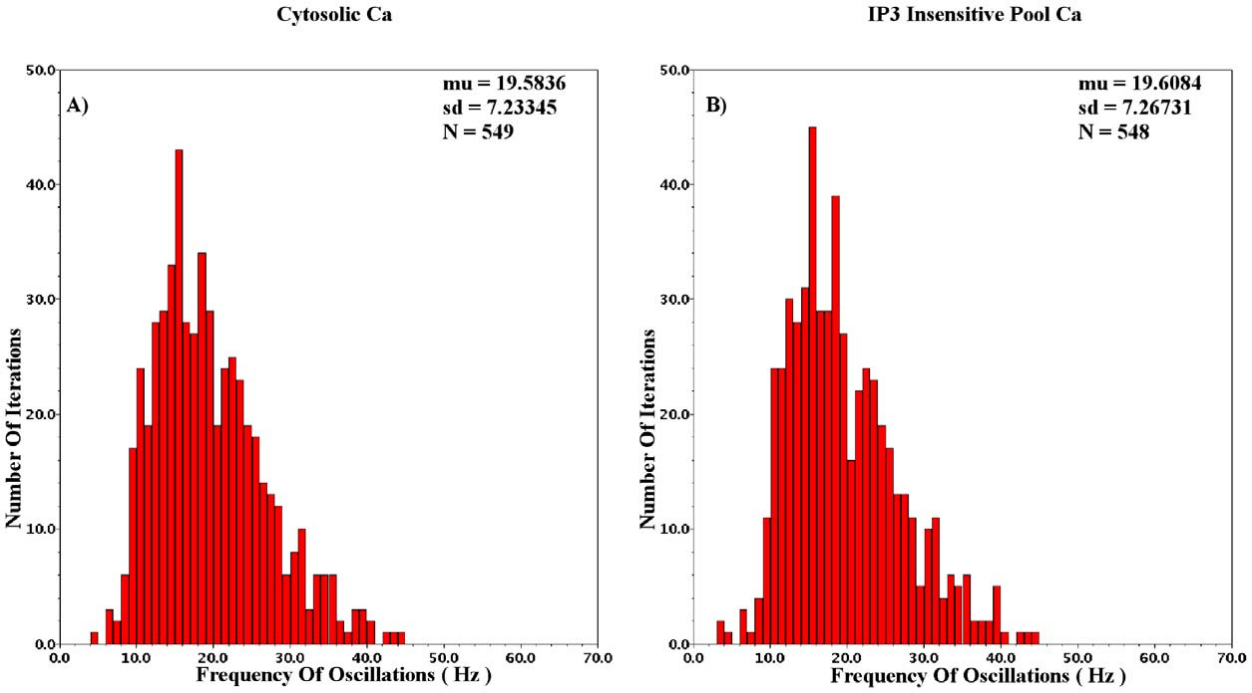
Monte Carlo Analysis for 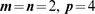 intracellular *Ca* ^2+^ Log-Domain circuit. 600 iterations have been performed, leading to a percentage greater than 90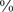 regarding successful oscillation runs.

**Figure 21:**
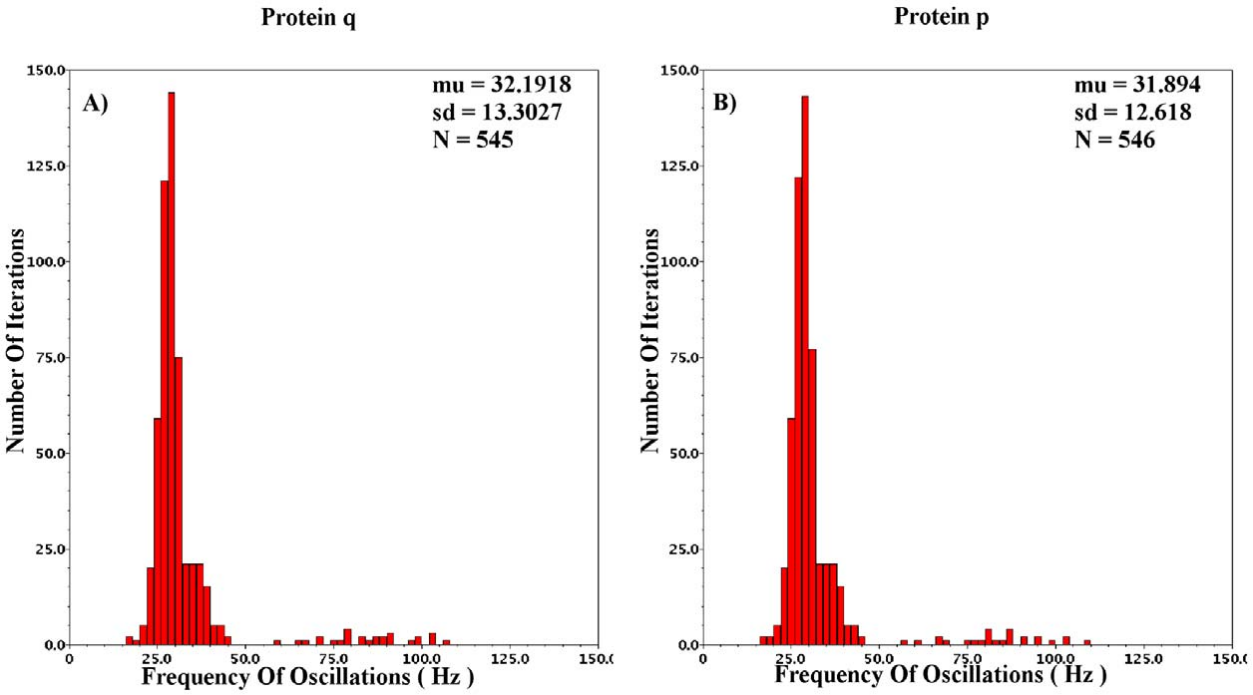
Monte Carlo Analysis for the two variable gene-protein regulatory Log-Domain circuit. Graphs A and B correspond to the various frequencies of protein q and p, respectively, throughout the analysis. 600 runs have been performed resulting to a successful percentage rate greater than 90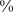.

**Figure 22:**
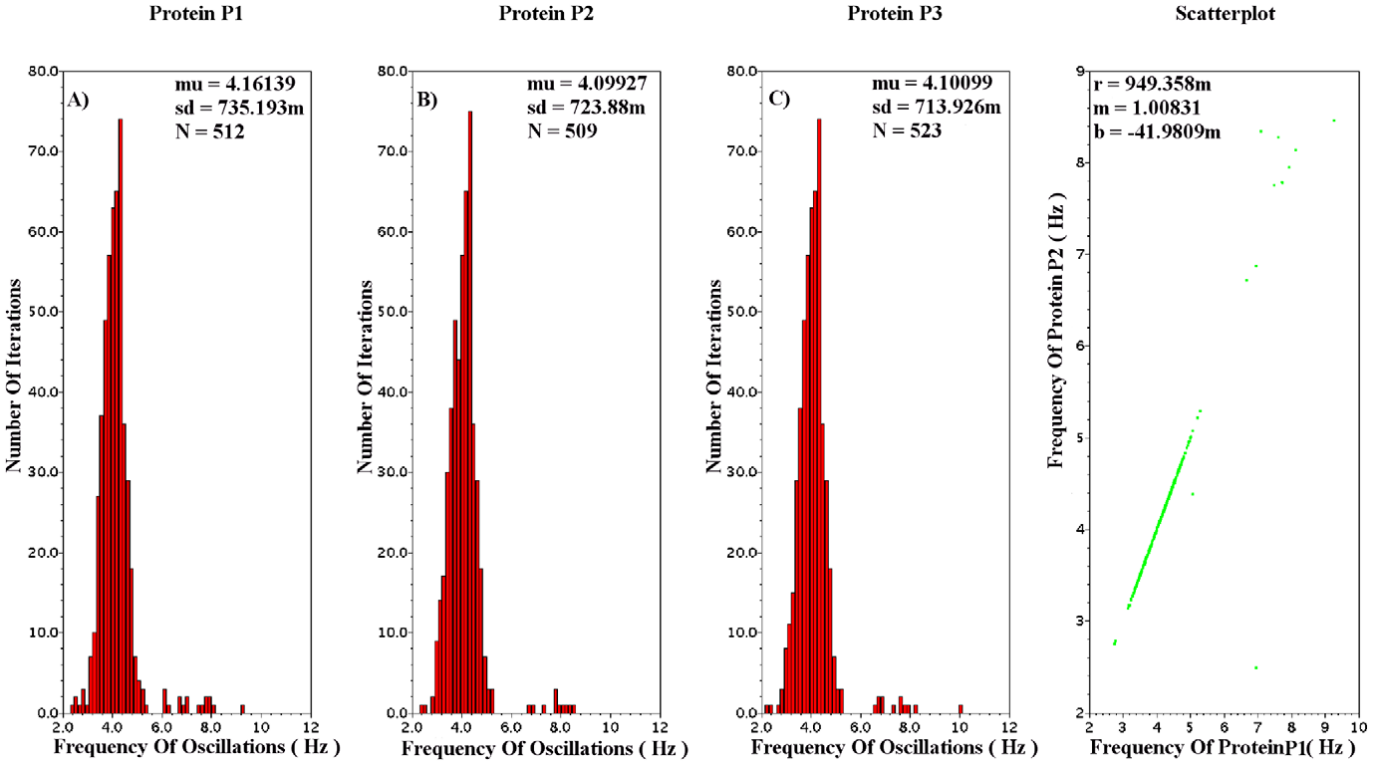
Monte Carlo Analysis for the three variable gene-protein regulatory Log-Domain circuit. Graph A corresponds to the various frequencies of oscillations of protein P1 during the 600 iterations of the analysis. Graphs B and C correspond to the various frequencies of proteins P2 and P3, respectively. The simulations have been performed for the current values presented in Tables 8 and 9. The number of successful oscillations is greater than 85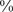.

The aim of CytoMimetic circuits is to emulate nonlinear biochemical dynamics, thus, their robustness is of great importance. The robustness of the proposed circuits has been assessed by means of Monte Carlo (MC) analysis. The output signals of the proposed circuits are the drain currents 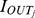 of each BC. Variations due to process and mismatch affect cumulatively such output currents. The MC analysis results presented in Figures 18, 19, 20, 21, and 22 demonstrate the number of successful oscillations for each output current versus the frequency of each oscillation, accompanied by their mean value and their standard deviation. Regarding the intracellular 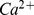 oscillations circuits, the 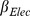 values that have been selected for the MC analysis of each model are the central ones (see Tables 4, 5, and 6). Since MC simulations generate a pool of data “*around*” a circuit's given operating point, it is vital to ensure that the simulated circuits' variations will be within the circuit's region of oscillation. Finally, in Table 9 an estimate of the proposed chips' area is demonstrated for the cases that the circuits' capacitors are built in and off chip. The capacitors are assumed to be POLY1-POLY2 (CPOLY) with CPOLY area capacitance 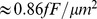.

Starting with the Log-Domain Intracellular 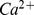 Oscillations circuits and more specifically with the 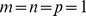 case, the MC analysis was performed for the values of Table 4 and 9, with 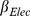 set equal to 0.55. The measured frequency for this value of 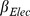 during transient analysis is 19.7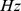. The mean MC frequency is 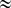
25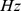 with standard deviation around 3.5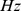. The adequate robustness of the specific circuit is accompanied by static power consumption close to 12.5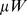 and approximate chip area of 0.5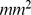.

In the 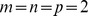 case the MC analysis was performed for the values of Tables 5 and 9 but with 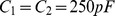 and aspect ratio for PMOS and NMOS devices set at 60/8 and 10/2, respectively. The 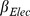 parameter was set at 0.7 and the frequency of oscillation for this value is 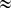
5.3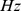. The mean value of the MC oscillations is 5.5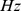 with standard deviation that approximates 2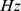. Again, the total chip size could be reduced by decreasing the total circuit capacitance which leads to slightly less similar dynamics. The total power consumption of this circuit is close to 6.5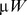, while the approximate chip area is 0.5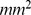.

The most “*sensitive*” version of the intracellular 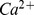 circuits, the 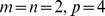 case has been tested for the values presented in Tables 6 and 9 but with 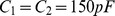 and the aspect ratio set at 17/8 and 8/1 for the PMOS and NMOS devices, respectively. The 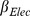 parameter was set at 0.4 leading to a sustained oscillation of frequency 19.8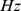. The mean MC frequency is 19.5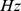 with standard deviation close to 7.2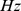. The total percentage of successful oscillations is higher than 90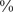. The chip area approximates 0.65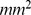 while the power consumed is close to 1.5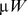. The various capacitance-aspect ratio combinations that have been adopted during MC analysis aim at highlighting the robustness of the proposed circuits, which are hardly affected by these factors.

The Log-Domain Gene - Protein Regulatory circuits have also been analysed by means of MC analysis. From the 2D case, the circuit implementing the 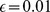 case has been chosen. Analysed for the values presented in Tables 7 and 9 the percentage of successful iterations is approximately 90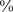. The mean frequency of the 600 MC runs is 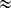
32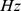 with standard deviation 12.5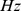 while the expected frequency for these values based on the transient analysis simulations is 27.5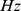. The circuit's static power consumption is approximately 1.3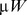 and its total chip area is close to 0.350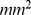. However, the circuit can emulate similar dynamics with 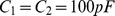 and minor changes of current values and aspect ratios.

The 3D category of the Log-Domain Gene - Protein Regulatory circuits also exhibits high percentages of successful oscillations in MC analysis. With an expected frequency of 4.9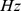, the circuit has been simulated for the values presented in Table 8 and 9 but with 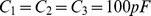 and aspect ratios 200/2 for both NMOS and PMOS devices. Similar MC results have been achieved for the capacitances and aspect ratios presented in Table 9. The mean MC frequency was approximately 4.1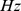 with the standard deviation being close to 0.7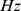. Finally, Figure 22 also illustrates a scatterplot for the frequencies of the successful oscillations of two proteins. The graph verifies that the points lie on a 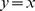 line, where 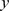 and 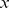 correspond to the various frequencies of the two proteins.

It is important to stress that although the proposed circuits have been tested for their robustness by means of the highly pessimistic MC analysis, the results obtained are adequately satisfactory. For very large VLSI cell networks the variability shown in the MC simulations is a feature that characterises CytoMimetic circuits, which implement the non-identical behaviour of multiple, real cellular responses [42], [43]. Real cells have variations and variations in the proposed circuits could mimic those, introducing biologically realistic randomness to the emulation.

### Effect of noise on CytoMimetic circuits

The noise behaviour of the presented topologies exhibits the basic characteristic on nonlinear logarithmic circuits operating in accordance with the large-signal exponential characteristic of the individual transistors, i.e. signal * noise intermodulation takes place. The case of Externally-Linear-Internally-NonLinear (ELIN), time-invariant responses has been studied both theoretically and by means of measurements and simulations [30],[44],[45]. It has been confirmed that when the input signal increases considerably in strength with respect to the input DC value (for example, in class-AB operation the ratio of these two quantities can be in the range of thousands), then the noise power increases with the power of the input.

The practical impact on performance of this “*signal-dependent noise floor*” behaviour is a saturated SNR ratio for high inputs. Hence, the performance of logarithmic and hyperbolic-sine ELIN responses is characterised by a high dynamic range under constant SNR for strong input signals. Transient Noise Analysis simulations performed on the novel CytoMimetic circuits studied here have confirmed the presence of signal * noise intermodulation. Though noise simulations are not presented due to lack of space, the interested reader can verify that the instantaneous noise tends to increase close to the peaks of strongly non-linear signals (e.g. the peak of the 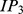 insensitive pool 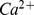 dynamics for the 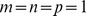 case in Figure 9 or the peak of Protein P1 dynamics in Figure 15) in direct analogy with the noise behaviour results presented in [30]. It would be useful to mention however that the robustness of the realised CytoMimetic behaviours does not seem to suffer when noise is taken into consideration.

## Discussion

In this paper, we have elaborated a systematic circuit synthesis method allowing for the direct mapping of nonlinear biological ODE models onto electrical circuits consisting only of transistors and capacitors and thus realisable by means of monolithic microchips. Such progress enables the implementation of a novel category of continuous-time, continuous-value VLSI biomimetic circuits, termed CytoMimetic circuits. Our design method is inspired by the Bernoulli Cell Formalism (BCF) used for the analysis and synthesis of dynamic translinear circuits. We have methodically modified the BCF formalism to yield a systematic electronic realisation method for nonlinear biochemical ODEs. The resulting electronic circuits provide ultra-low-power, fast and accurate means of simulating or predicting cellular or molecular nonlinear dynamics. Simulated results of novel circuit topologies mimicking the nonlinear dynamics of (a) an intracellular calcium oscillations model and of (b) a gene-protein regulatory system model have been used to illustrate the detailed method.

CytoMimetic circuits for cellular/molecular dynamics computation have a plethora of possible or envisioned future applications. Firstly, such circuits open up the possibility of efficiently simulating the dynamical responses of large networks of cells or even of accurately mimicking the behaviour of small tissues or organs. Indeed, based on such technology, the molecular dynamics of large numbers of interconnected biological systems can be efficiently simulated in real-time in silico by a microchip with minute power demands and relatively small size. Secondly, when coupled to arrays of biosensors and bioactuators, CytoMimetic circuits can form the basis of fast and relatively cheap, reusable high-throughput drug testing platforms or, alternatively, be employed for the robust and optimal control of biological systems (either natural systems or synthetic biology engineered systems). Both of the aforementioned applications have been investigated by few researchers based on microchips designed using approaches that, contrary to NBCF, do not rely on explicitly defined relationships between the electrical and biological variables. We therefore anticipate that VLSI analog CytoMimetic chips, in principle, have the potential to provide a more efficient and rigorous solution to the applications outlined above.

All of the aforementioned represent only a minor part of the potential applications that ultra-low-power biocircuits can have an impact on. It is highly likely that future developments exploiting the methods presented here will shed even more light on the range of applications that such circuits can enable, revealing a promising path for further fruitful research in cybernetic electronics.
